# Mitochondria Pathway Signature Predicts Prognosis and Therapeutic Response and Identifies REXO2 as a Crucial Regulator in Breast Cancer

**DOI:** 10.1155/mi/8994064

**Published:** 2026-02-16

**Authors:** Zizhao Guo, Heng Cao, Chuqi Lei, Dongxu Ma, Jiang Wu, Zeyu Xing, Chenyu Zhao, Xiang Wang, Jianxiu Cui

**Affiliations:** ^1^ Department of Breast Surgical Oncology, National Cancer Center/National Clinical Research Center for Cancer/Cancer Hospital, Chinese Academy of Medical Sciences and Peking Union Medical College, Beijing, 100021, China, cacms.ac.cn; ^2^ Department of Breast Surgery, Beijing Chaoyang Hospital Affiliated to Capital Medical University, Beijing, 100020, China, bjcyh.com.cn; ^3^ School of Pharmacy, Queen’s University Belfast, Belfast, BT9 7BL, UK, qub.ac.uk

**Keywords:** breast cancer, immunotherapy, inflammatory microenvironment, mitochondrial pathway, prognosis

## Abstract

**Background:**

Mitochondrial‐related pathways (MRPs) play a crucial role in cancer metabolism and progression; however, their prognostic value in breast cancer (BC) is still poorly understood.

**Methods:**

We integrated multiomics data to investigate the landscape of MRPs in BC. A mitochondria pathways‐associated signature (MPAS) was established using multimachine learning framework and interpreted by SHAP analysis across independent BC cohorts. Additionally, a series of functional experiments were employed to explore the role of RNA exonuclease 2 (REXO2) in BC cells.

**Results:**

MRPs are extensively activated in BC at multiomics level. MPAS demonstrates outstanding predictive performance across multiple BC cohorts, with high scores indicating poor clinical outcomes. Moreover, it was observed that high MPAS scores are closely associated with immunosuppressive states and inflammatory microenvironments. SHAP analysis identified REXO2 as a hub factor of MPAS. Cell‐based work confirmed that silencing REXO2 greatly inhibited cell proliferation and induced apoptosis in BC.

**Conclusions:**

Our proposed MPAS could effectively evaluate the prognosis and treatment response of BC patients, providing new reference for clinical decision‐making. Furthermore, REXO2 regulates cell proliferation and apoptosis, making it a promising potential therapeutic target for inhibiting BC progression.

## 1. Introduction

Breast cancer (BC) is the most common cancer among females worldwide and a leading cause of death in women [1]. As the leading cause of cancer incidence and mortality among women globally, BC is estimated to reach 4.8 million new cases by 2050 [2]. Current treatment strategies for BC include local therapy (surgery and radiotherapy) and systemic therapy (chemotherapy and targeted therapy), with treatment selection guided by disease stage and molecular pathogenesis [3]. Although remarkable advances have been achieved in BC, the global mortality rate for this disease remains high [4–6]. Therefore, it is urgent to detect the potential mechanisms in BC progression and develop novel therapeutic targets.

Mitochondria, as key organelles in cellular energy metabolism, exert crucial roles in numerous biological processes, including reactive oxygen species (ROS) balance, cell death, and lipid transport [7]. Growing evidence indicates that mitochondrial function is critical in tumor progression and immune regulation [8–10]. For example, enhanced mitochondrial biogenesis promotes tumorigenesis through regulation of glycolytic metabolism in multiple tumors, including hepatocellular carcinoma (HCC) and esophageal cancer [11, 12]. Moreover, mitochondrial oxidative phosphorylation (OXPHOS) has been reported to be closely associated with tumor stemness, metastasis, and treatment failure in multiple types of tumors [13–15]. For instance, Deng et al. [16] uncovered that TACO1 facilitates bladder cancer stemness and drug resistance by enhancing OXPHOS. Additionally, mitophagy promotes mitochondrial homeostasis by regulating metabolic reprogramming and balancing the clearance of damaged mitochondria, which in turn supports tumor survival [17–19]. As revealed by Sun et al. [20], targeting BNIP3 could inhibit melanoma development by blocking mitophagy and OXPHOS. Moreover, it has been shown that ANT3‐mediated mitophagy contributes to the progression of multiple myeloma and treatment failure [21]. In summary, these studies provide new insights for developing targeted mitochondrial therapies in tumor. However, the function role of diverse mitochondrial‐related pathways (MRPs) on BC progression and their prognostic value remain poorly understood.

Through comprehensive multiomics analysis, we detected mitochondrial pathway activity and clinical implications in BC. Machine learning framework was applied to develop mitochondria pathways‐associated signature (MPAS), a MRP‐associated signature for evaluating clinical outcomes and therapeutic response. By integrating clinical data from multiple independent BC cohorts, we validated the stability and reliability of MPAS in assessment of prognosis. Further detection of MPAS indicated that RNA exonuclease 2 (REXO2) is a key gene in this signature. Experimental works further clarified that REXO2 could affect BC progression by regulating cell proliferation and apoptosis.

## 2. Materials and Methods

### 2.1. Data Acquisition

Gene expression profiling and clinical information for BC were downloaded from The Cancer Genome Atlas (TCGA). Moreover, three independent BC cohorts (GSE20685, GSE42568, and GSE58812) from the Gene Expression Omnibus (GEO) were incorporated as validation sets. The BC dataset (GSE176078) for single‐cell analysis was collected from the GEO database. We obtained a list of 149 MRPs and 1136 associated protein‐coding genes from the MitoCarta3.0 database (https://www.broadinstitute.org/mitocarta/). Detailed information for 149 MRPs is presented in Supporting Information Table S1.

### 2.2. Evaluation of MRPs in BC

MRP activity for each BC sample was evaluated by using single‐sample gene set enrichment analysis (ssGSEA). Log2‐scaled expression matrices were used as input together with curated pathway gene sets supplied as a GMT object.

### 2.3. Single‐Cell Analysis

Single‐cell analysis was conducted using the Seurat package [22]. We then selected the top 2000 highly variable genes via the FindVariableFeatures function. FindNeighbors and FindClusters functions were applied to perform clustering analysis. The resulting cell populations were visualized using Uniform Manifold Approximation and Projection (UMAP). Annotation of cell clusters into malignant, immune, and stromal lineages was performed by evaluating the expression of canonical marker genes. To investigate cell–cell interactions, we applied the CellChat package to infer ligand‐receptor communication networks and probabilities among the identified cell types.

### 2.4. Spatial Transcriptomic (ST) Data Analysis

ST data for BC were obtained from the official website of 10x Genomics which was processed in Python using Scanpy [23]. Quality control was performed to exclude low‐quality spots and genes, followed by normalization and log transformation of the expression matrix. Highly variable features were then selected to support subsequent dimensionality reduction, clustering analyses, and data visualization.

### 2.5. Establishment of MPAS in BC

Univariate Cox analysis was employed to screen candidate genes with significant prognostic value in BC. Subsequently, we employed a multialgorithmic approach to build the optimal prognostic model. A total of 10 different algorithms were used, including stepwise Cox, Lasso, Ridge, plsRcox, CoxBoost, random survival forest (RSF), generalized boosted regression, elastic net, supervised principal components, and survival support vector machine. Model optimization was performed via 10‐fold cross‐validation, and the best‐performing model was selected based on the highest concordance index (C‐index). Detailed methods are presented in the Supplementary Methods.

### 2.6. SHAP Interpretable Analysis

We applied SHAP analysis to assess the contribution of each gene to the prognostic model using the R packages kernelshap and shapviz. The multivariate Cox model was fitted on the training dataset, and additive SHAP values were computed based on gene expression features, with the mean absolute SHAP value used to rank gene importance. No additional feature selection threshold was applied during SHAP estimation.

### 2.7. Immunotherapy Analysis

Differences of immune checkpoint gene expression between the two MPAS groups were analyzed using boxplot plots. Immune, stromal, and ESTIMATE scores of BC patients were computed using the “estimate” R package. To evaluate potential responsiveness to immunotherapy, Immunophenoscores (IPS) for TCGA‐BRCA samples were obtained from The Cancer Immunome Atlas (TCIA) database, and IPS differences between two MPAS groups were subsequently compared.

### 2.8. Cell Culture

Human normal breast epithelial (MCF10A) and BC (MDA‐MB‐468, MDA‐MB‐231, and MCF7) cell lines were obtained from the Cell Bank of the Chinese Academy of Sciences in Shanghai, China. All cell lines were recently authenticated and confirmed to be free of mycoplasma contamination. All cells were cultured in DMEM containing 10% fetal bovine serum (FBS; Gibco) and 1% penicillin‐streptomycin. Cultures were maintained in a humidified incubator at 37 °C with a 5% CO_2_ supply.

### 2.9. Quantitative Real‐Time Polymerase Chain Reaction (qRT‐PCR)

Total RNA was extracted using the TRIzol reagent (Thermo Fisher) and reverse‐transcribed into cDNA from 1 µg RNA using the PrimeScript RT Reagent Kit (Takara). qRT‐PCR was performed with SYBR Green PCR Master Mix (Thermo Fisher) on aStepOnePlus Real‐Time PCR System (Thermo Fisher). Gene expression was normalized to β‐actin and quantified using the 2^−ΔΔCt method. Primer sequences are provided in Supporting Information Table S2.

### 2.10. Cell Transfection and Lentivirus Construction

Lentiviral vectors encoding REXO2, an empty control, shRNA targeting REXO2 (sh‐REXO2), and a nontargeting control (sh‐NC) were purchased from GenePharma (Shanghai, China). For transduction, cells were seeded and infected at ~50% confluency. Stably transduced cells were subsequently selected using puromycin (Sigma–Aldrich) for 7 days. The sequences for the sh‐REXO2 constructs are listed in Supporting Information Table S3.

### 2.11. Cell Counting Kit‐8 (CCK‐8) Assay

To measure cell proliferation, a CCK‐8 assay (Beyotime Biotechnology) was performed. Cells were plated in 96‐well plates at 3000 cells per well. Cell viability was measured daily from day 1 to day 5. At each time point, 10 μL of CCK‐8 reagent was added to each well and incubated for 2 h at 37 °C. The optical density (OD) was then measured at 450 nm using a microplate reader.

### 2.12. EdU Assay

Cells were seeded in 6‐well plates (2 × 10^5^ cells/well) and cultured for 24 h, followed by incubation with 50 μM EdU for 2 h. After fixation with 4% paraformaldehyde and permeabilization with 0.5% Triton X‐100, EdU incorporation was detected using Apollo reaction reagent, with nuclei counterstained by Hoechst 33342 (or DAPI). Cells were subsequently analyzed by flow cytometry.

### 2.13. Flow Cytometric Analysis

Cellular apoptosis was assessed by flow cytometry using an Annexin V‐FITC and propidium iodide (PI) staining kit (Vazyme Biotech) per the manufacturer’s protocol. Following detachment, cells were resuspended in 1x binding buffer and stained with Annexin V‐FITC (5 µL) and PI (5 µL) for 15 min at room temperature, protected from light. Stained samples were immediately analyzed on a flow cytometer.

### 2.14. Statistical Analysis

All bioinformatics analyses were conducted using R software (version 4.1.0). The statistical analysis was analyzed by GraphPad Prism 10. Student’s *t*‐test was employed for comparisons between two groups. A *p*‐value < 0.05 was considered statistically significant.

## 3. Results

### 3.1. Characterization of MRPs in BC

**Figure 1:**
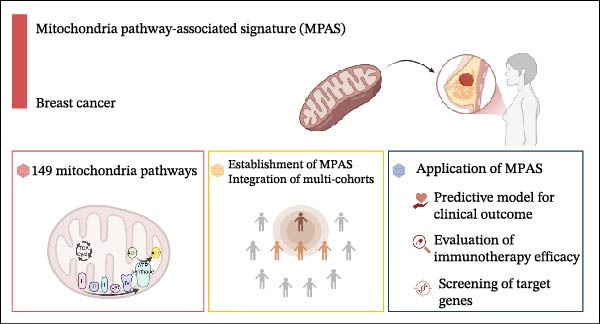
The overall workflow of the present study.

The overall workflow of the present study is shown in Figure 1.

To comprehensively investigate the involvement of MRPs in BC, we first compared the activity of 149 MRPs between BC and normal tissues using data from TCGA, GEO, and GTEx databases. Pathway activity for this comparison was quantified using ssGSEA analysis. It was observed that most MRPs were activated in tumor samples, including OXPHOS, nucleotide metabolism, and fatty acid oxidation. Conversely, pathways involved in apoptosis, mtRNA stability and decay, and mitochondrial permeability transition pore (MPTP) were markedly suppressed in tumor cohorts (Figure 2A). Next, the landscape of MRPs was detected on a BC single‐cell dataset. A total of 98,656 cells with six major cell types were identified after performing quality control and cell annotations (Figure 2B,C). Interestingly, similar results were observed by using ssGSEA algorithm to evaluate the activity of MRPs in each cell of BC single‐cell data (Figure 2D). In addition, we assessed MRP activities in BC ST data. As shown in Figure 2E, pathways such as OXPHOS, nucleotide metabolism, and fatty acid oxidation were greatly enriched in tumor regions, whereas almost no signal was observed of pathways related to apoptosis, mtRNA stability and decay, and MPTP. In summary, these findings suggest that MRPs may exert a critical role in BC development.

Figure 2Characterization of mitochondria‐related pathways (MRPs) in BC. (A) Landscape of the activity of different MRPs in different BC cohorts. (B) Uniform Manifold Approximation and Projection (UMAP) plots showing six major cell types in BC. (C) Dotplot displaying the marker genes across different cell types. (D) Landscape of the activity of different MRPs in different cell types. (E) Representative spatial transcriptomic (ST) section of BC tissue. (Top left) H&E staining of the same section. (Bottom left) Spatial localization of annotated tumor cells. (Right panels) Spatial activity maps of representative MRPs, including oxidative phosphorylation (OXPHOS), nucleotide metabolism, fatty acid oxidation, apoptosis, mtRNA stability and decay, and mitochondrial permeability transition pore (MPTP).

**Figure:**
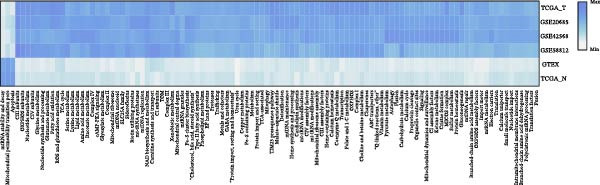
(A)

**Figure:**
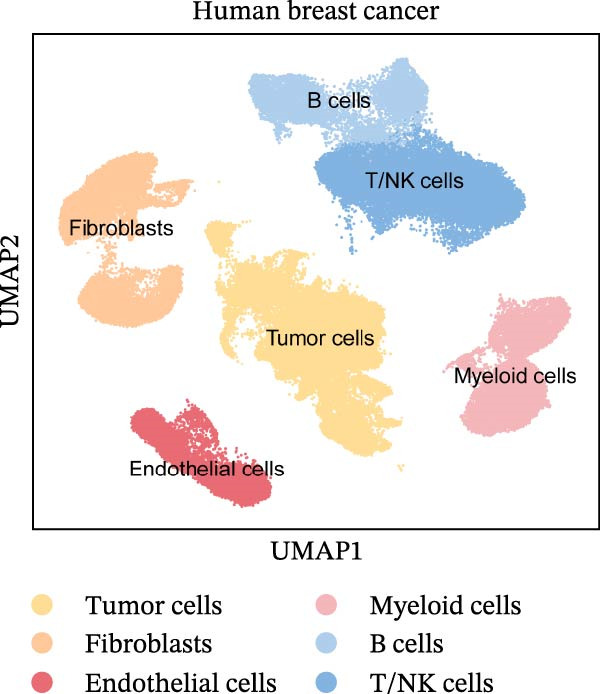
(B)

**Figure:**
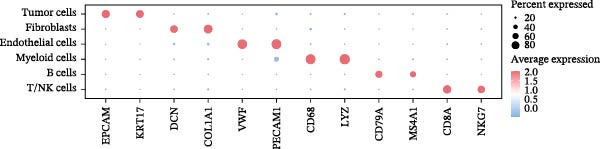
(C)

**Figure:**
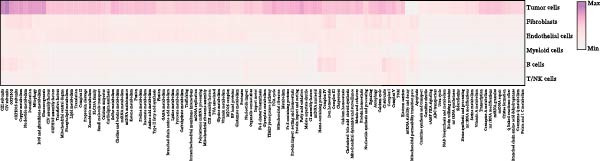
(D)

**Figure:**
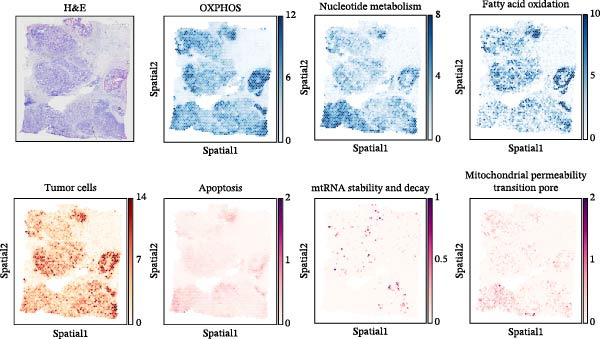
(E)

### 3.2. Establishment of MPAS in BC

To construct a favorable MPAS model, univariate Cox analysis was first applied to screen 86 potential MRP genes with significant prognosis value in the TCGA cohort (Supporting Information Table S4). Then, 10 machine learning methods were employed to generate an optimal predictive model. Based on the average C‐index values, RSF + Enet (alpha = 0.2) with the highest AUC value (0.733) was selected for MPAS development (Figure 3A). Subsequently, SHAP algorithm was applied to evaluate the contribution of feature genes to the MPAS on predictive performance. Based on the ranking of mean SHAP values, the top five features (REXO2, CASP9, GSTK1, HSPA9, and VDAC1) were identified as the hub genes of MPAS model (Figure 3B). Violin plots demonstrated the relationship between each feature value and its corresponding SHAP value (Figure 3C), where larger SHAP values indicate a greater impact on the predictive performance. Additionally, both the force plot and waterfall plot determined REXO2 as a central component of the MPAS (Figure 3D,E). Furthermore, we detected the clinical relevance of top features in MPAS by using HPA website and GEPIA2 database (Figure S1).

Figure 3Establishment of mitochondria pathways‐associated signature (MPAS) in BC. (A) The optimal model identified by machine learning heatmap selection was employed as the MPAS scoring system. (B) Bar plot showing the mean absolute SHAP values of the top five features contributing to the MAPS model. (C) Bee‐swarm plot displaying the distribution of SHAP values for individual samples. Each point represents a single patient, with color indicating the feature value (orange, high; purple, low). Features with higher SHAP values exerted a stronger positive effect on risk prediction. (D) Force plot illustrating the direction and magnitude of each feature’s contribution to an individual patient’s prediction. (E) Waterfall plot summarizing the cumulative effects of the top contributing features on the overall prediction output, highlighting the dominant role of REXO2.

**Figure:**
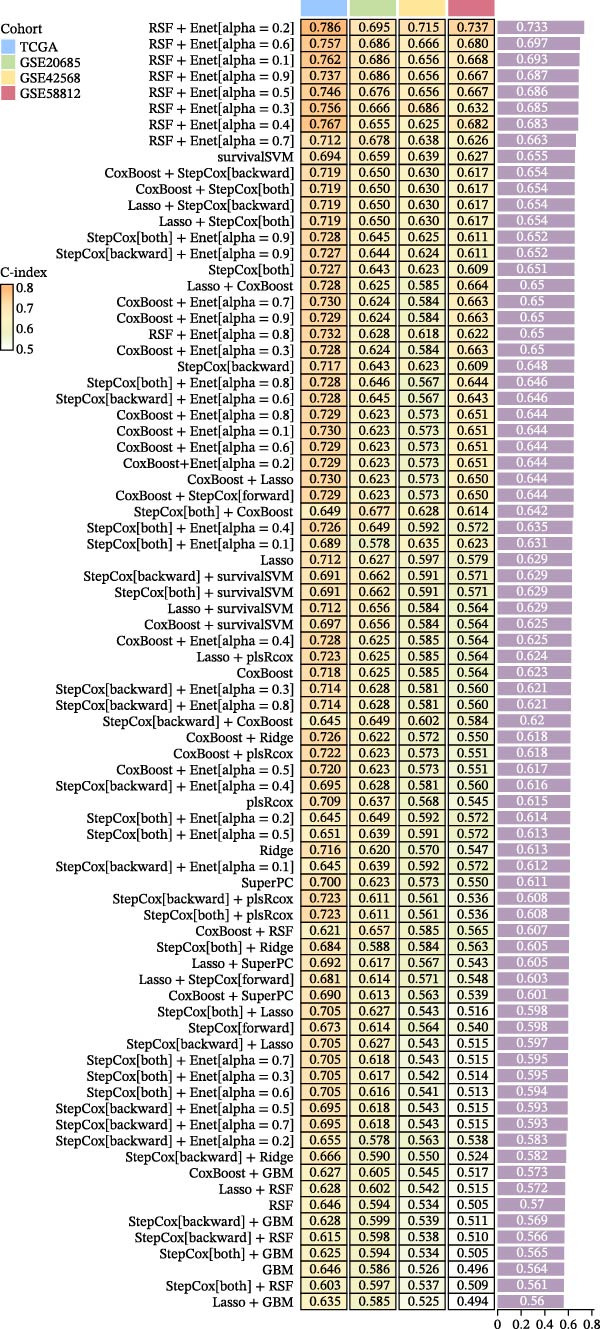
(A)

**Figure:**
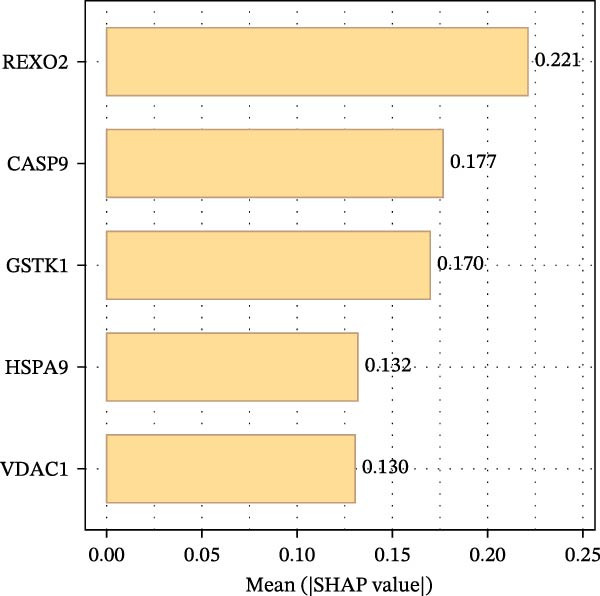
(B)

**Figure:**
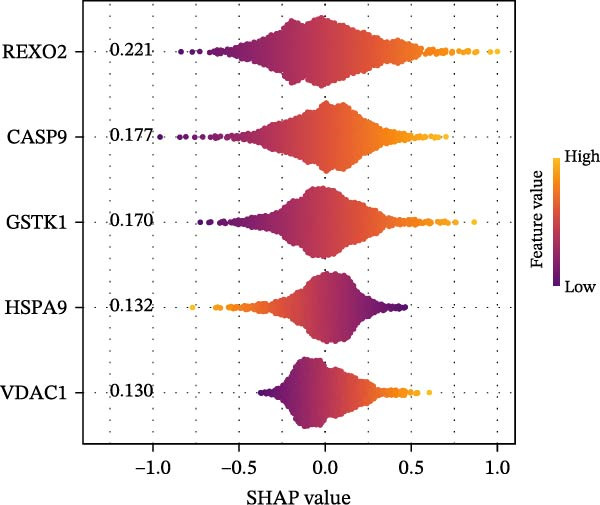
(C)

**Figure:**
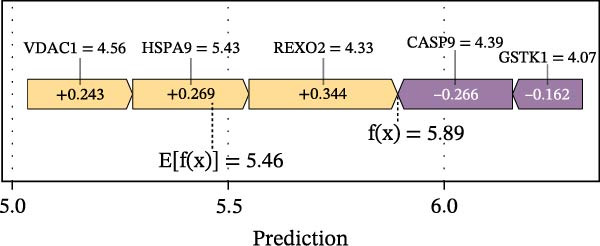
(D)

**Figure:**
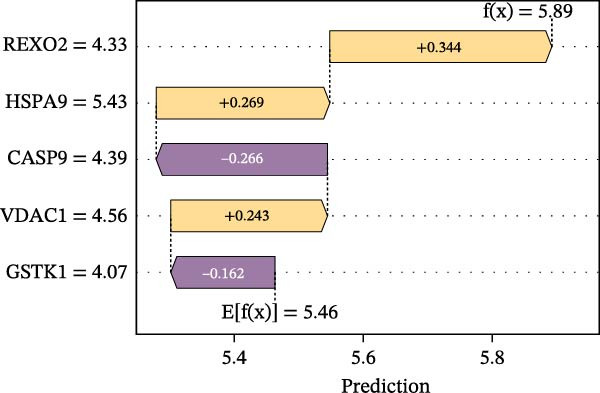
(E)

### 3.3. Validation of the MPAS Prognostic Performance

To confirm the prognostic performance of our proposed MPAS, all BC cases were divided into high‐ and low‐MPAS subgroups based on the median MPAS score. Survival analyses demonstrated that patients with high MPAS scores exhibited remarkably poor outcomes in the training set. Also, similar results were obtained across three independent cohorts (Figure 4A). ROC curves disclosed stable predictive accuracy, with the AUC values reaching 0.814, 0.815, and 0.875 at 1, 3, and 5 years, respectively, in the TCGA cohort. Comparable predictive performance was observed in the independent cohorts, including GSE20685 (AUC: 0.751–0.883), GSE42568 (AUC: 0.833–0.890), and GSE58812 (AUC: 0.789–0.863) (Figure 4B). Moreover, the distributions of MPAS score revealed a clear prognostic difference between the two MPAS groups (Figure 4C).

Figure 4Validation of the MPAS prognostic performance. (A) Survival analysis of MPAS in multicohorts including TCGA, GSE20685, GSE42568, and GSE58812. (B) ROC curves showing predictive performance of MPAS across cohorts. (C) Distribution of risk scores and corresponding survival time and status of patients in each cohort.

**Figure:**
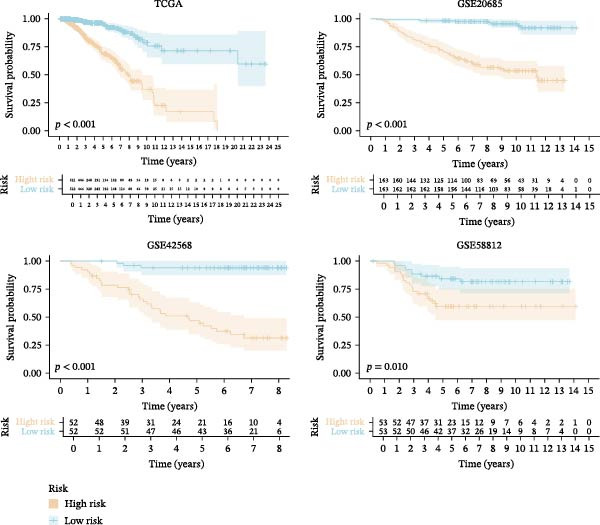
(A)

**Figure:**
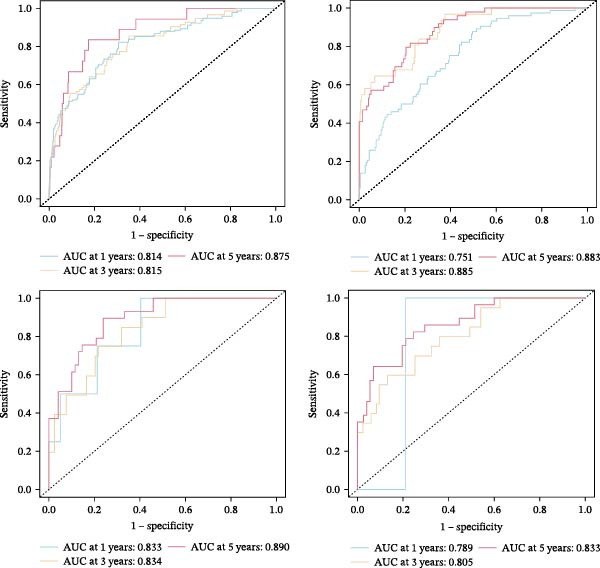
(B)

**Figure:**
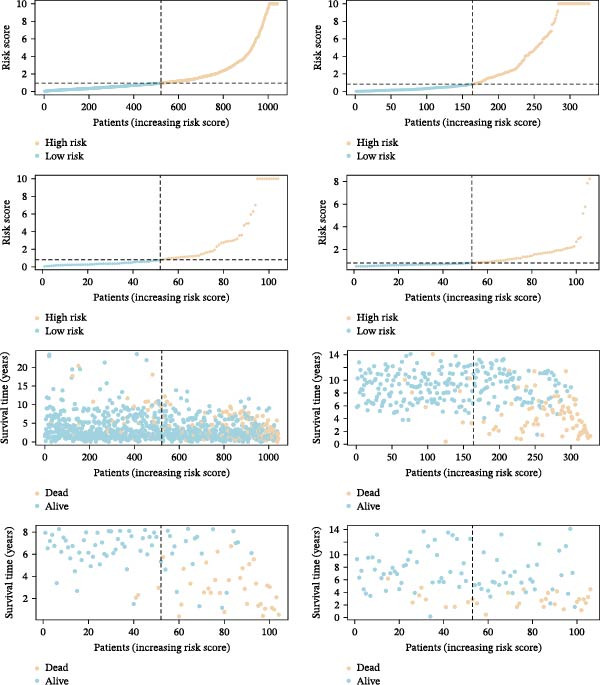
(C)

Subsequently, we applied Cox regression methods to detect the independent prognostic value of MPAS. It was shown that MPAS score remained an independent prognostic factor for OS after performing multivariate Cox analysis (Figure 5A,B). We next evaluated the prognostic performance of MPAS in different clinical subgroups including stage, T stage, and N stage. Survival curves demonstrated that MPAS still displayed a robust performance in predicting outcomes in different clinical subgroups (Figure 5C–H).

Figure 5Independent prognostic and subgroup analyses of MPAS. Univariate (A) and multivariate (B) Cox regression analyses were used to examine the independent prognostic value of MPAS in the TCGA dataset. Subgroups survival analyses of MPAS across different clinical subgroups: stage (C–D), T stage (E–F), and N stage (G–H).

**Figure:**
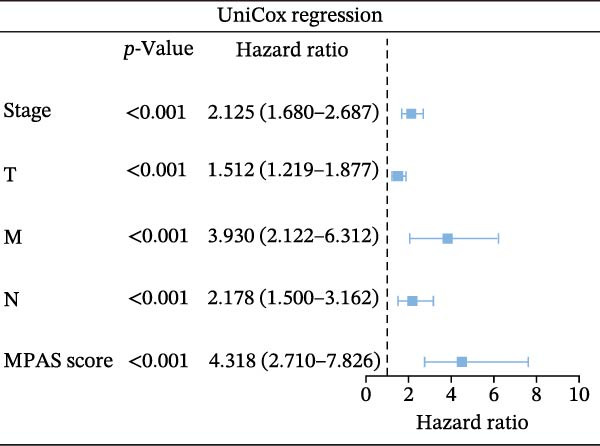
(A)

**Figure:**
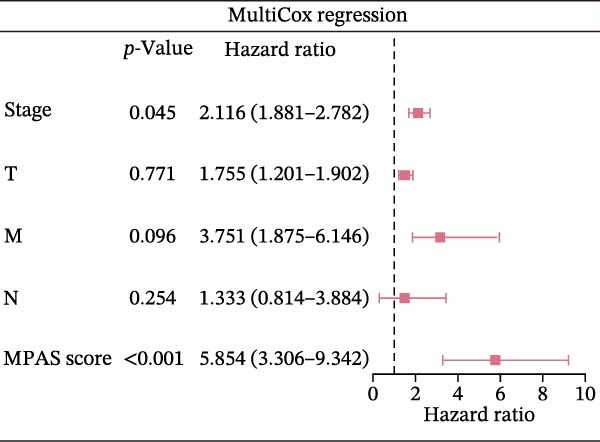
(B)

**Figure:**
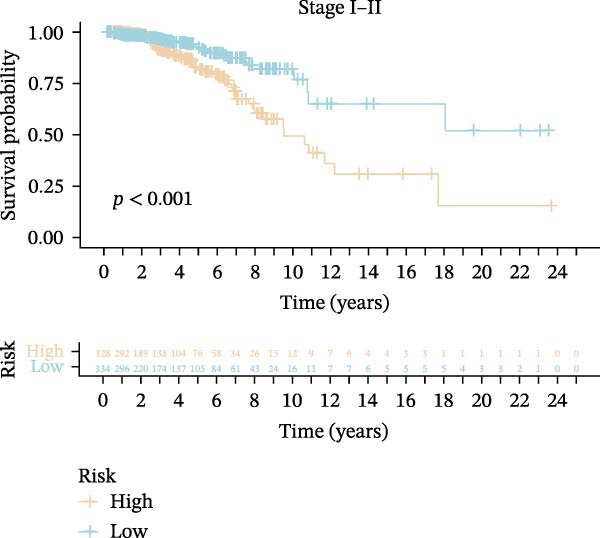
(C)

**Figure:**
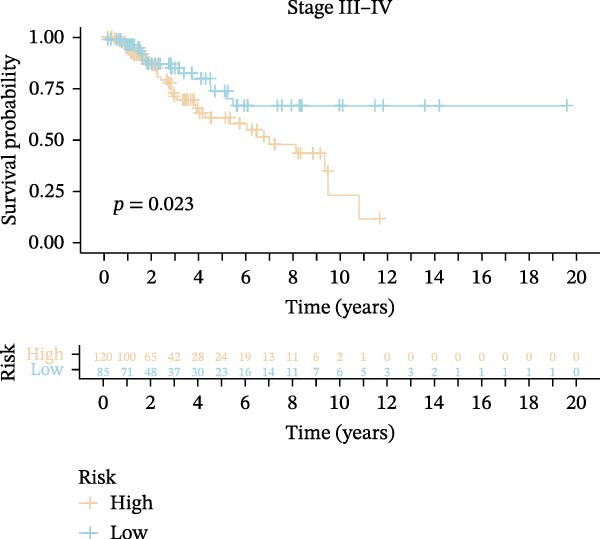
(D)

**Figure:**
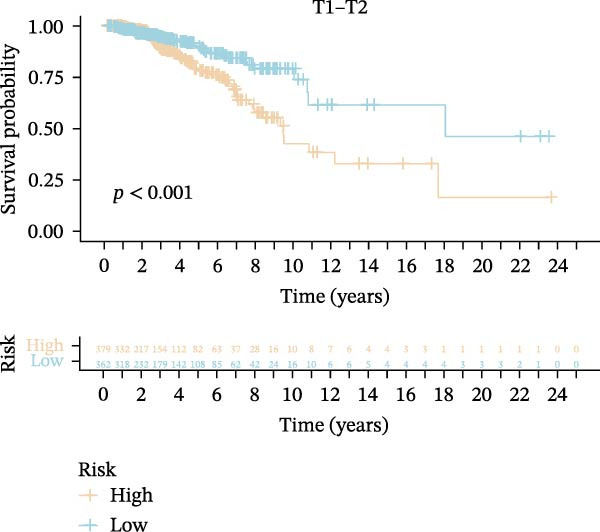
(E)

**Figure:**
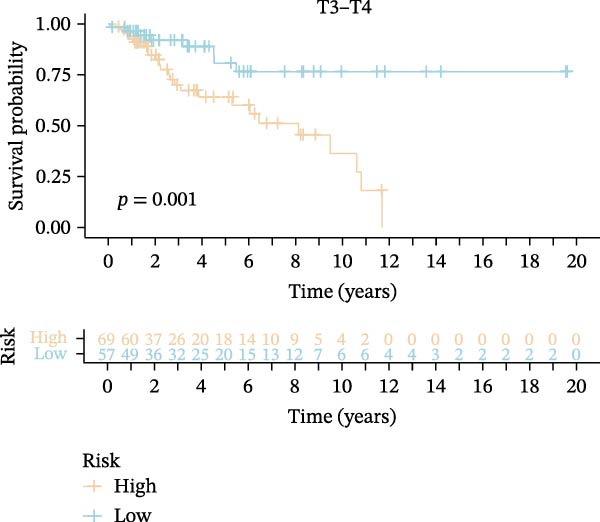
(F)

**Figure:**
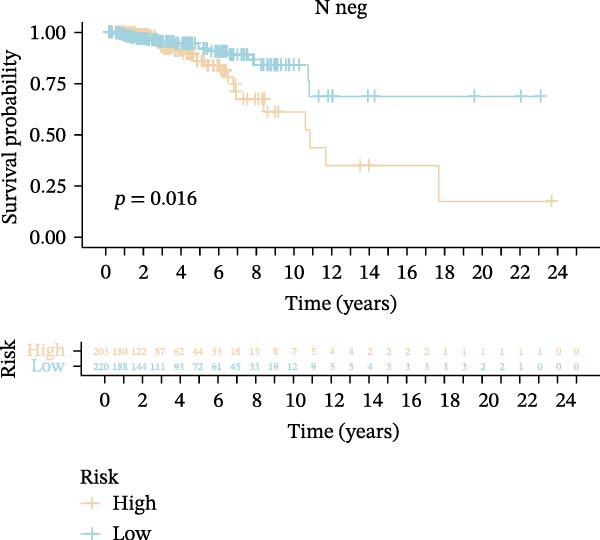
(G)

**Figure:**
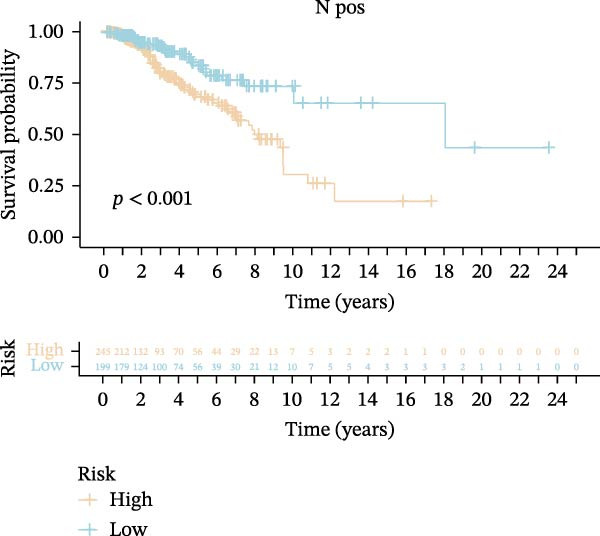
(H)

### 3.4. Correlation Analysis Between MPAS and Tumor Immune Landscape

Systematic detection of immune characteristics disclosed distinct patterns between MPAS subgroups. Immune function analysis revealed that T cell coinhibition, checkpoint expression, and inflammatory promotion were highly enriched in the high‐MPAS group (Figure 6A). We then estimated the immunocyte infiltration of each BC patient. As suggested by Figure 6B, patients with high MPAS scores presented a decreased infiltration of antitumor lymphocytes, including CD8^+^ T cells, and B cells, and a higher infiltration of immunosuppressive components, such as M2 macrophages. Notably, we determined upregulation of hub costimulatory genes such as CD70 and CD80 in the low‐MPAS group (Figure 6C). Furthermore, we detected the expression of classical immune checkpoints between the two MPAS groups (Figure 6D). To explore the relationship between MPAS and inflammatory status, we compared the expression patterns of inflammation‐related genes (IRGs) between the two MPAS groups. As shown in Figure 6E, we found upregulation of multiple IRGs in the high‐MPAS group. Moreover, the inflammatory score was positively correlated with the MPAS score (Figure 6F).

Figure 6Correlation analysis between MPAS and tumor immune landscape. (A) Radar plots of immune function characteristics between two MPAS groups. (B) Correlation between the MPAS and immune cell infiltration evaluated by different computational algorithms (CIBERSORT, EPIC, MCP‐counter, QUANTISEQ, TIMER, and xCell). (C) Heatmap of immune‐related gene expression. (D) Expression profiles of immune checkpoints between two MPAS groups. (E) Expression profiles of inflammation‐related genes between two MPAS groups. (F) Correlation analysis of inflammatory score and MPAS.

**Figure:**
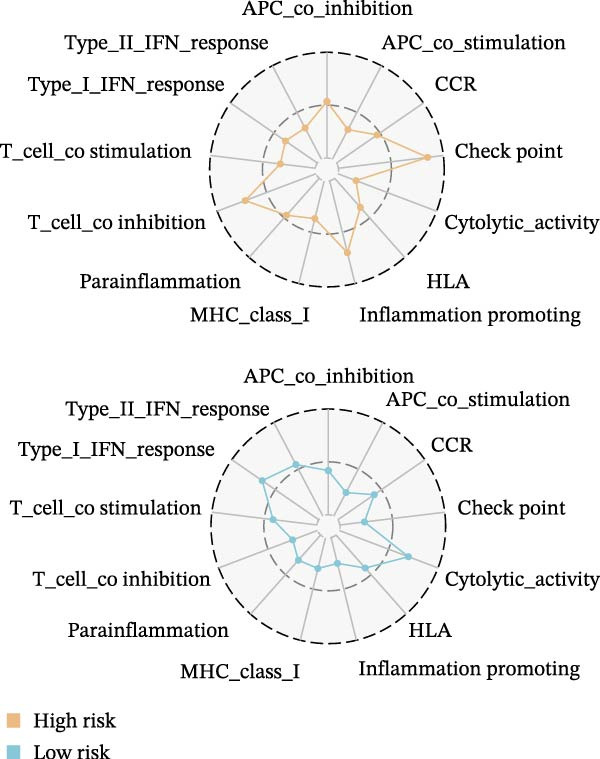
(A)

**Figure:**
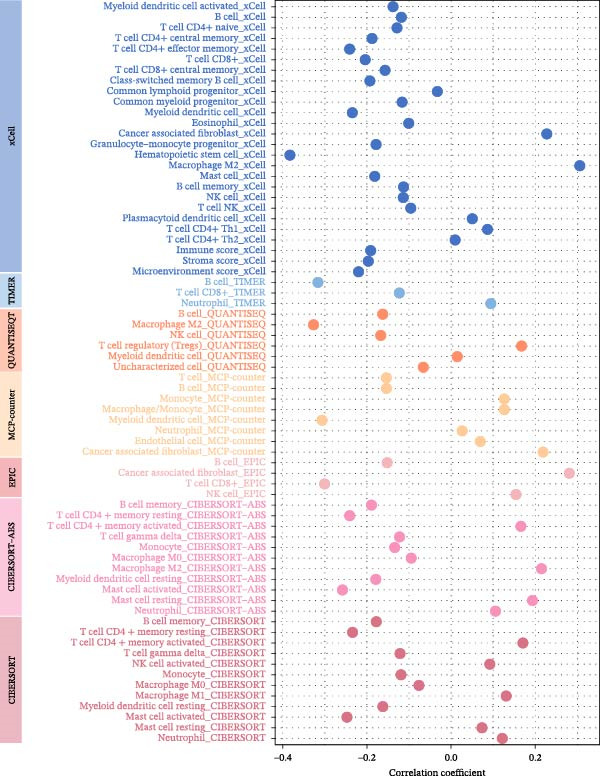
(B)

**Figure:**
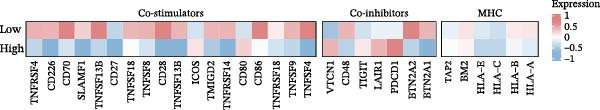
(C)

**Figure:**
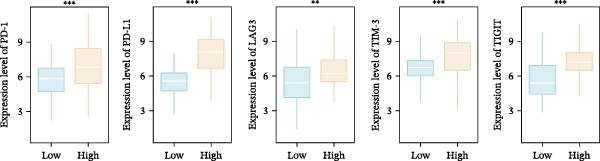
(D)

**Figure:**
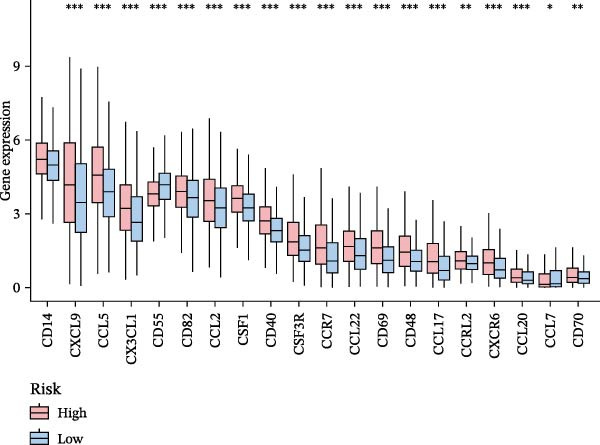
(E)

**Figure:**
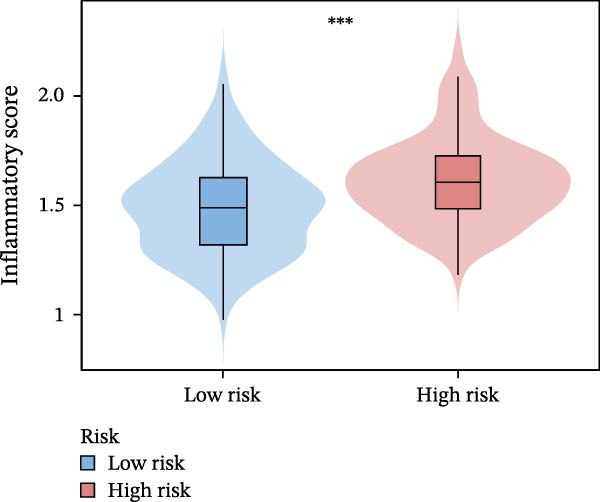
(F)

### 3.5. Association Between MPAS and Immunotherapy Efficacy

Subsequently, the relationship between MAPS and immunotherapy response was detected. As revealed by Figure 7A, patients with low MAPS scores presented a higher tumor mutational burden (TMB) value. Survival analysis indicated that BC cases with low TMB value showed poor clinical outcome (Figure 7B). It was observed that the low‐MAPS/high‐TMB subgroup showed favorable prognosis (Figure 7C). Moreover, it was observed that antigen presentation, immune cell migration, T cell infiltration, and tumor cell recognition were greatly enriched in the low‐MAPS group (Figure 7D). In contrast, immunosuppressive signatures such as immune checkpoints, MDSCs, and Tregs were greatly enriched in the high‐MAPS group (Figure 7E). TIDE analysis indicated that the high‐MPAS group was characterized by higher T cell dysfunction and immune exclusion (Figure 7F–H). In addition, patients in the low‐MPAS group presented higher IPS scores, indicating that they could obtain greater benefit from immune checkpoint blockade (ICB) therapy (Figure 7I–L).

Figure 7Correlation analysis between MPAS and tumor immune landscape. (A) Comparison of tumor mutational burden (TMB) between two MPAS groups. (B) Survival analysis illustrating that patients with high TMB had significantly better overall survival compared with those with low TMB. (C) Combined survival analysis integrating both MAPS and TMB levels. (D) Radar plots of cancer immunity cycle characteristics between two MPAS groups. (E) Analysis of immunosuppressive signatures between two MPAS groups. Correlation between MAPS and immune escape scores including (F) TIDE, (G) dysfunction, and (H) exclusion scores. (I–L) Immunophenoscores (IPS) analysis for MPAS.

**Figure:**
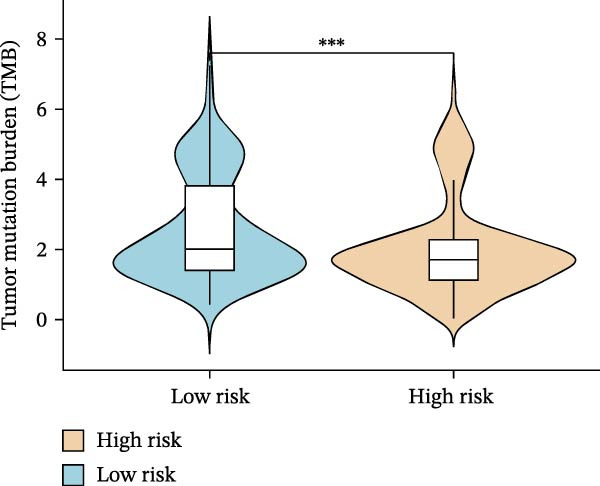
(A)

**Figure:**
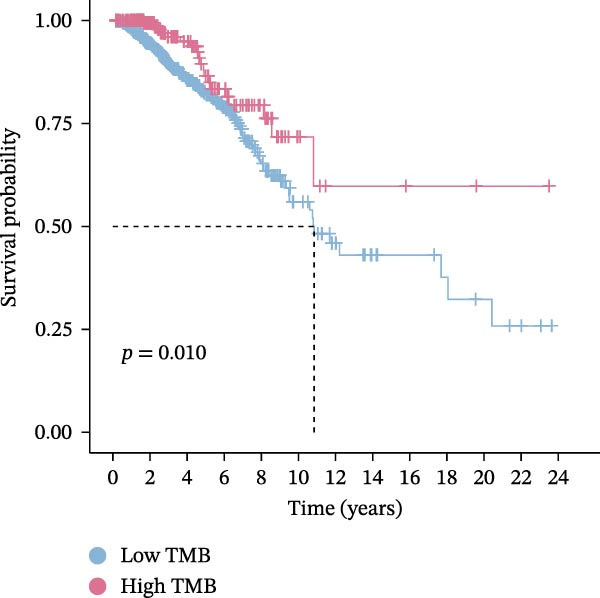
(B)

**Figure:**
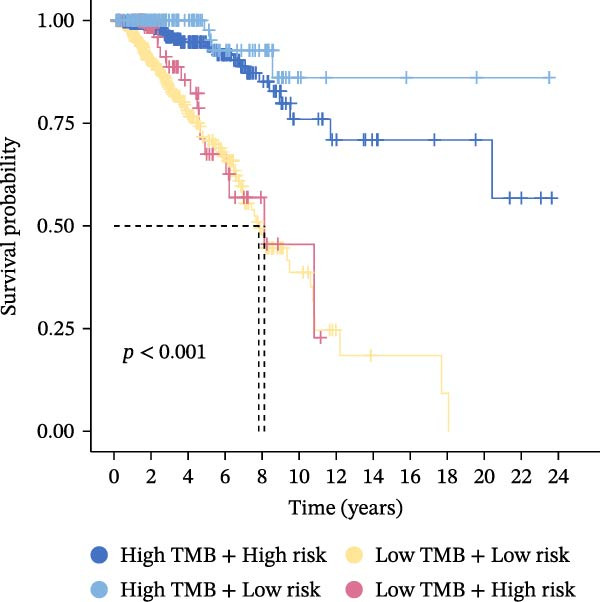
(C)

**Figure:**
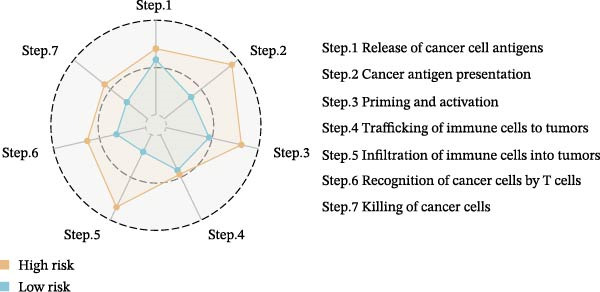
(D)

**Figure:**
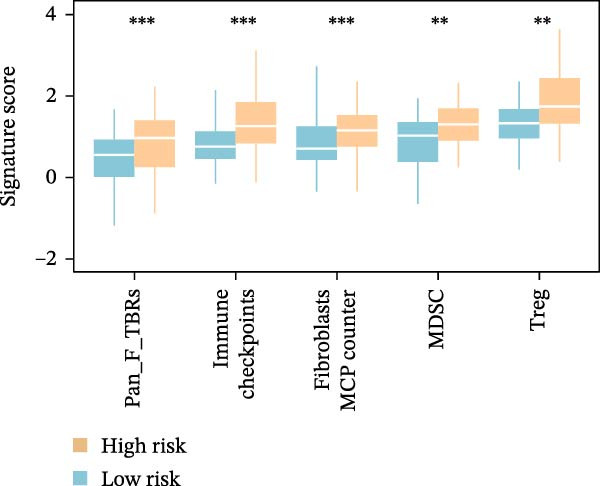
(E)

**Figure:**
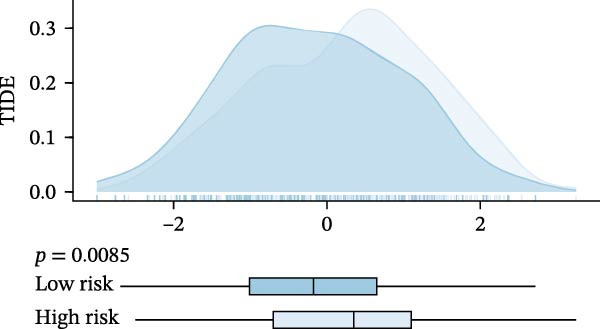
(F)

**Figure:**
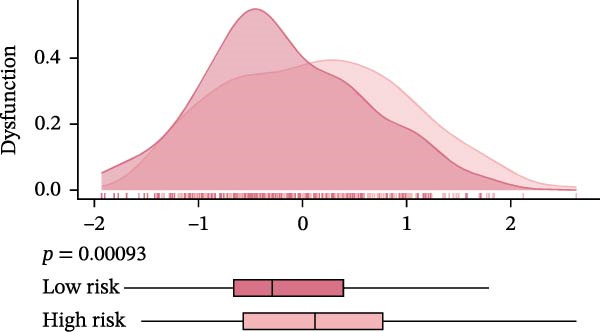
(G)

**Figure:**
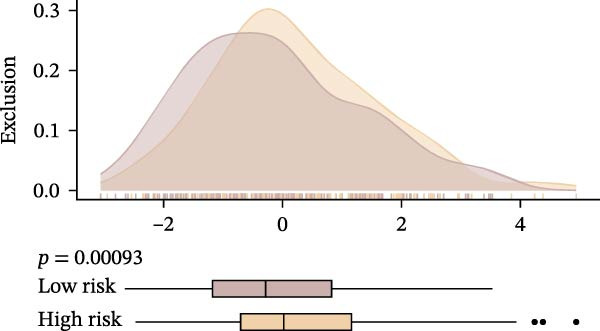
(H)

**Figure:**
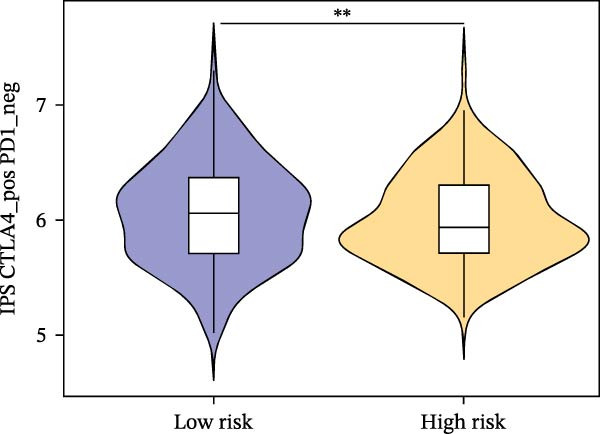
(I)

**Figure:**
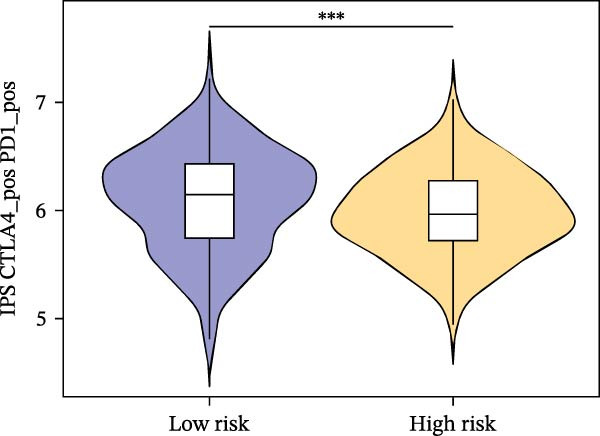
(J)

**Figure:**
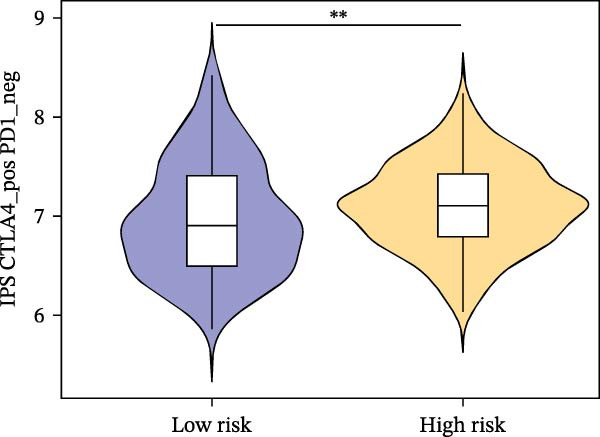
(K)

**Figure:**
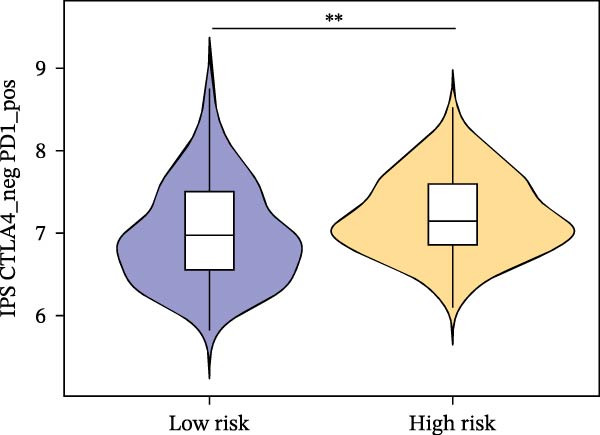
(L)

### 3.6. Single‐Cell Landscape of MPAS in BC

To uncover the role of the MPAS at single‐cell level, all cells were stratified into high‐MPAS and low‐MPAS subgroups based on the median MPAS score (Figure 8A–C). It was observed that high‐MPAS group had a higher proportion of tumor cells (Figure 8D). Also, violin plots demonstrated that tumor cells had the highest MPAS scores compared to other cell populations (Figure 8E). Then, we applied CellChat analysis to compare cellular interactions between the two MPAS groups. It was revealed that high‐MPAS cells exhibited greatly more cell‐to‐cell interactions (Figure 8F). Specifically, tumor cells displayed stronger communication with fibroblasts and endothelial cells in the high‐MPAS group (Figure 8G,H). Pathway signaling analysis demonstrated that the high‐MPAS group was characterized by the significant activation of MDK and CDH1 signaling (Figure 8I). We next confirmed the spatial distribution patterns of key ligand‐receptor pairs (MDK/SDC1 and CDH1/ITGB1) on BC ST data. Figure 8J demonstrates that the interactions were mainly enriched in the tumor regions.

Figure 8Single‐cell landscape of MPAS in BC. (A) UMAP plots of annotated cell types in BC. (B) Distribution of MPAS scores. (C) MPAS group classification dividing cells into high‐ and low‐MPAS groups. (D) Bar plot of cell proportions in two MPAS groups. (E) Violin plots of MPAS score distribution across cell types. (F) Quantification of intercellular communication between two MPAS groups. (G) Cell communication networks in two MPAS groups. (H) Heatmaps of cellular interaction strengths in two MPAS groups. (I) Differential signaling pathway analysis. (J) Spatial distribution patterns of hub ligand‐receptor pairs on BC slides.

**Figure:**
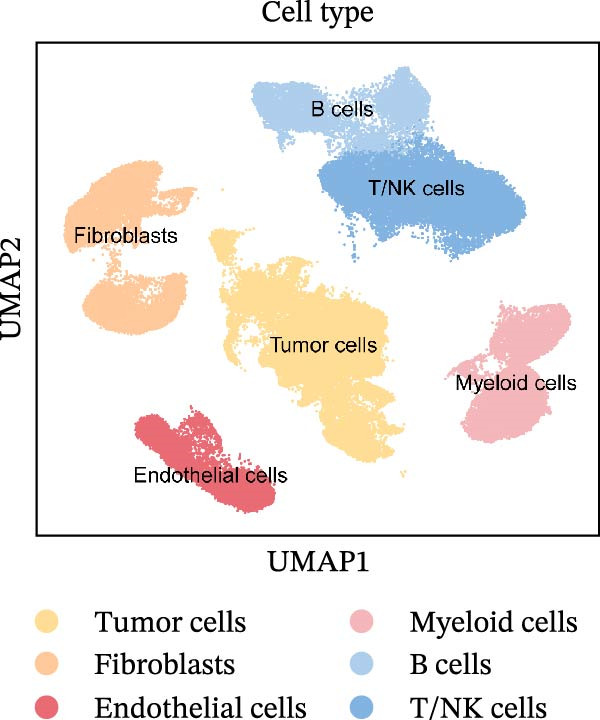
(A)

**Figure:**
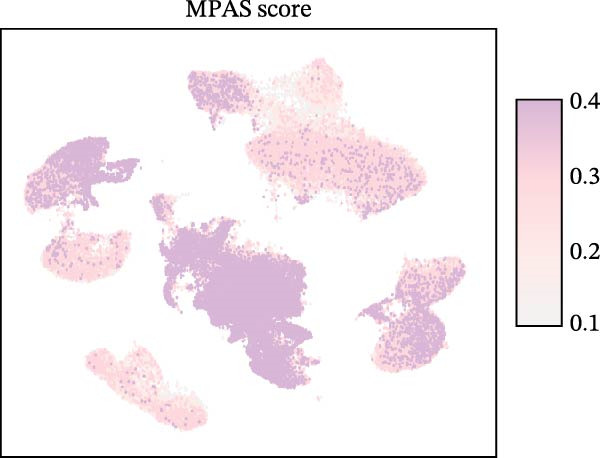
(B)

**Figure:**
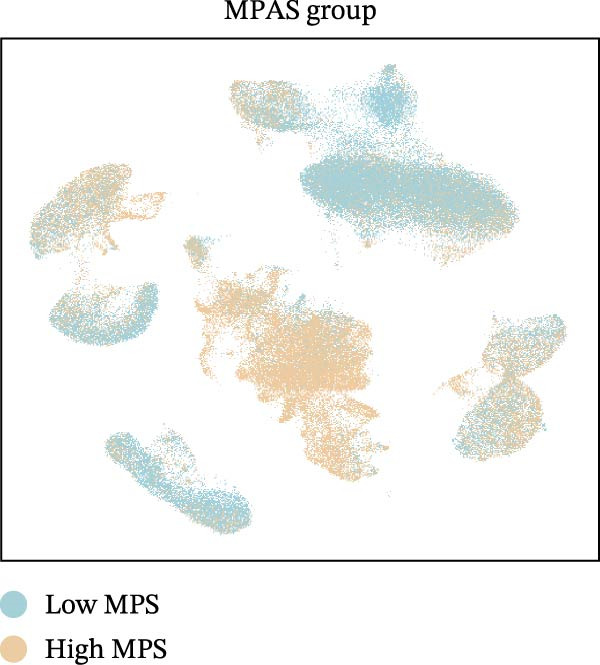
(C)

**Figure:**
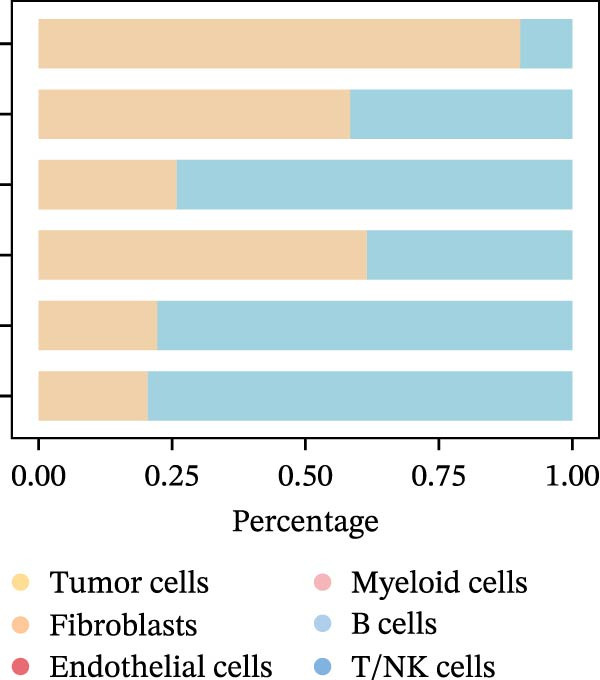
(D)

**Figure:**
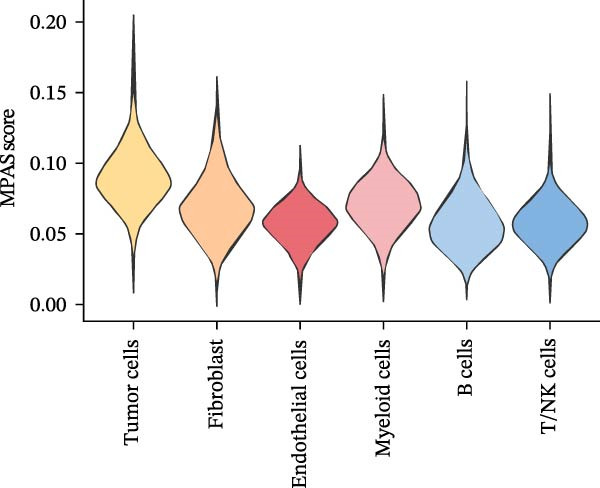
(E)

**Figure:**
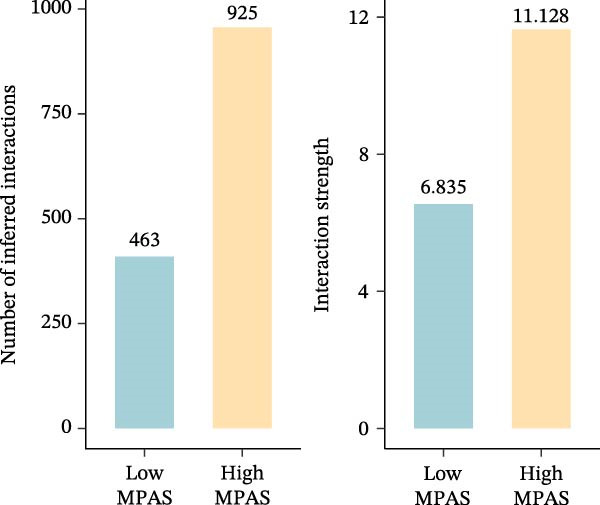
(F)

**Figure:**
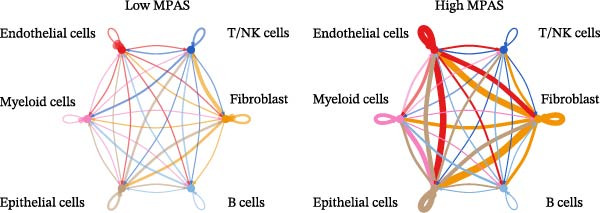
(G)

**Figure:**
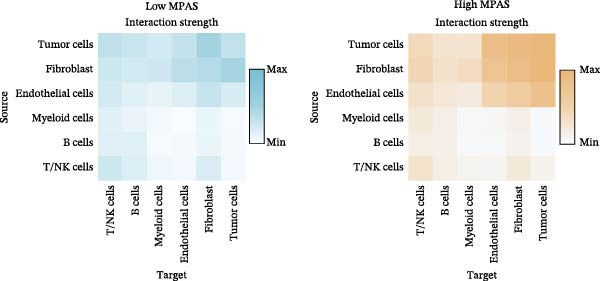
(H)

**Figure:**
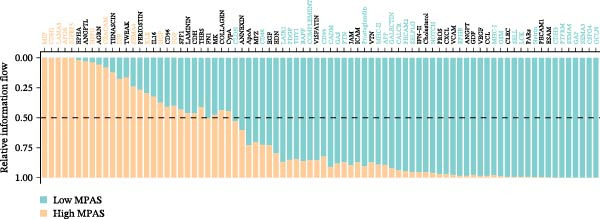
(I)

**Figure:**
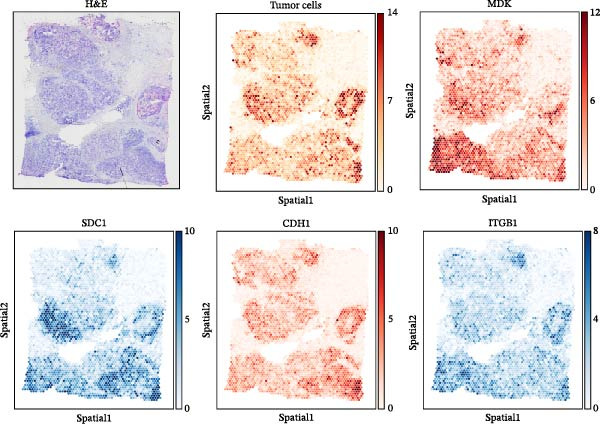
(J)

### 3.7. Function Enrichment Analysis

We next employed the GSEA method to uncover the relationship between potential function and MPAS. Notably, inflammatory response, glycolysis, and WNT signaling were enriched in the high MPAS group, whereas several hallmarks including apoptosis, DNA repair, and p53 pathway were involved in the low MPAS group (Figure S2).

### 3.8. Drug Sensitivity Analysis

To further explore the potential therapeutic implications of MPAS, we applied the oncoPredict package to estimate drug sensitivity. It was found that patients in the low‐MPAS group exhibited greatly higher sensitivity to several chemotherapeutic and targeted agents, including GSK269962A, cisplatin, docetaxel, gefitinib, and P22077 (Figure S3A–E). In contrast, patients in the high‐MPAS group were more sensitive to lapatinib, rapamycin, and 5‐fluorouracil (Figure S3F–H).

### 3.9. Exploration of the Functional Role of REXO2 in BC Progression

Based on the clues provided by the SHAP analysis, REXO2 was determined as a key contributor of MPAS. As shown in Figure 9A, immunohistochemistry results from the HPA website suggested markedly upregulation of REXO2 in BC tissues. We next detected the expression pattern of REXO2 across different cell lines and observed that REXO2 was highly expressed in BC cell lines compared with normal breast epithelial cells (Figure 9B). Subsequently, favorable transfection efficiency was confirmed by qRT‐PCR assay (Figure 9C,D). To determine the functional role of REXO2 in BC cell proliferation, we performed CCK‐8 and EdU assays. It was found that silencing REXO2 greatly suppressed proliferation in MDA‐MB‐231 cells (Figure 9E,G). Conversely, overexpression of REXO2 markedly promoted proliferation in MDA‐MB‐468 cells (Figure 9F,H). Furthermore, we investigated the effect of REXO2 on apoptosis using flow cytometry. The results demonstrated that REXO2 knockdown could induce apoptosis of BC cells (Figure 9I–K), while the opposite results were observed in the overexpression group (Figure 9J‐L).

Figure 9Exploration of the functional role of REXO2 in BC progression. (A) Immunohistochemical staining of REXO2 protein in BC and normal tissues from HPA database. (B) Expression pattern of REXO2 in different cell lines. (C–D) Transfection efficiency in BC cell lines detected by qRT‐PCR assay. (E–F) CCK‐8 assays in MDA‐MB‐231 and MDA‐MB‐468 cells with REXO2 knockdown or overexpression, respectively. (G–H) Flow cytometric analysis of EdU. (I–L) Flow cytometric analysis of apoptosis.

**Figure:**
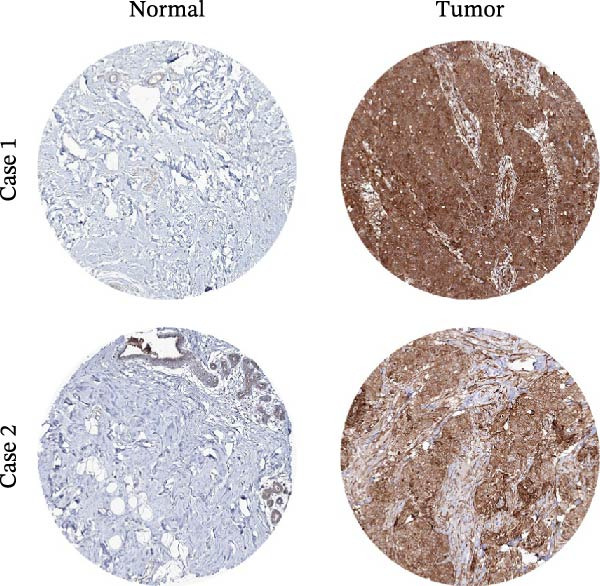
(A)

**Figure:**
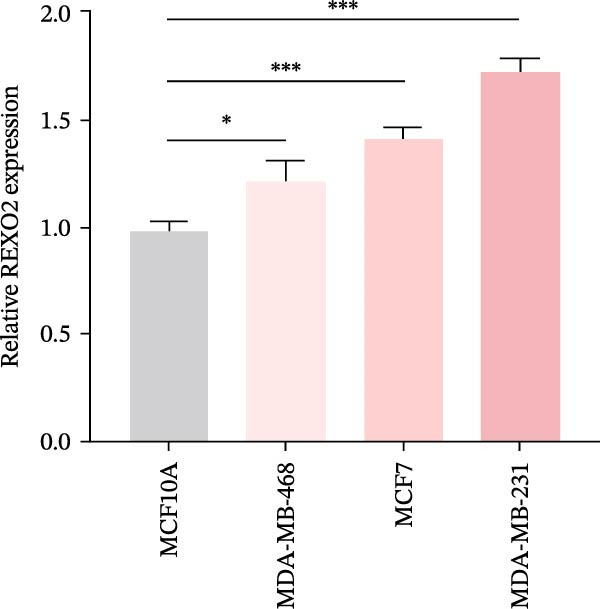
(B)

**Figure:**
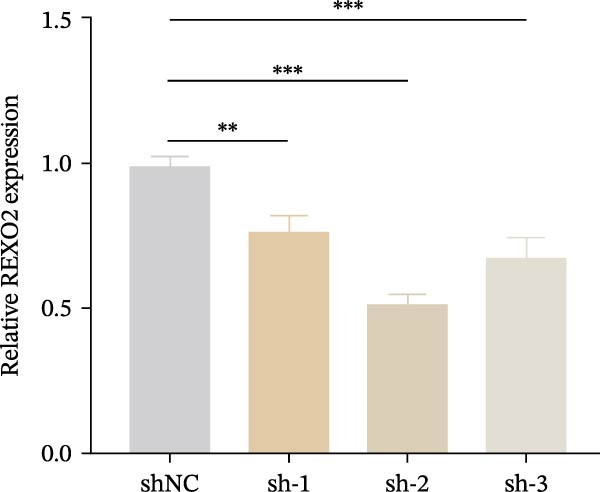
(C)

**Figure:**
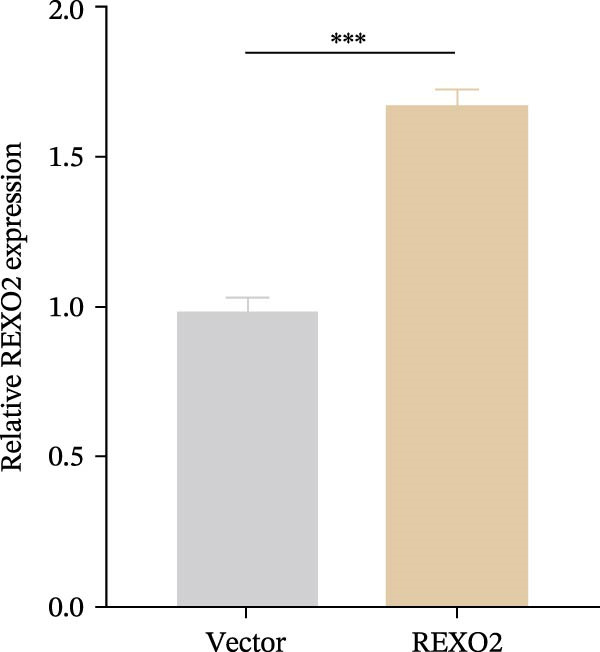
(D)

**Figure:**
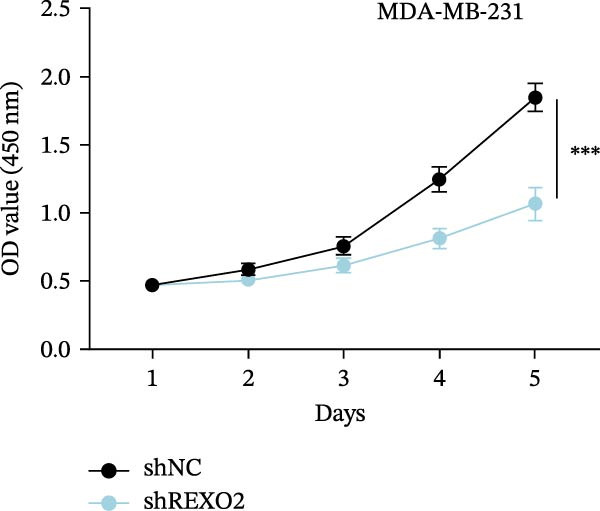
(E)

**Figure:**
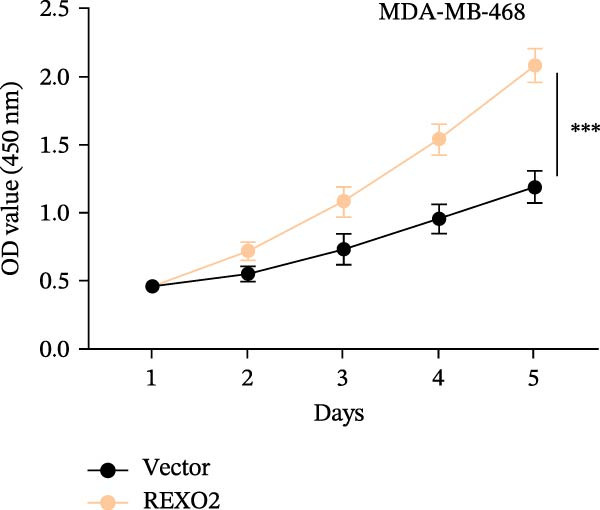
(F)

**Figure:**
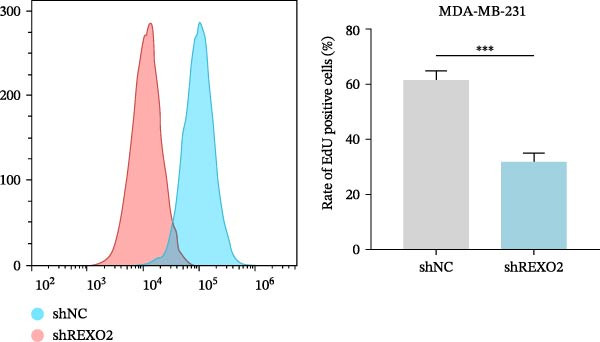
(G)

**Figure:**
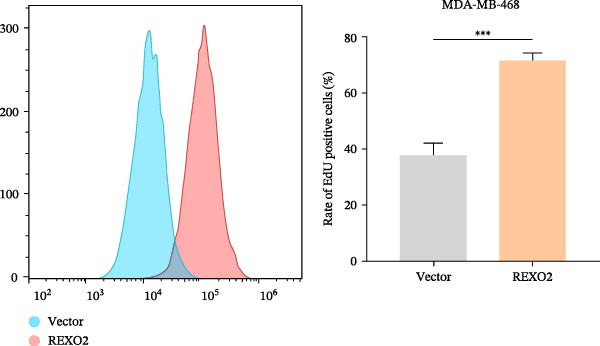
(H)

**Figure:**
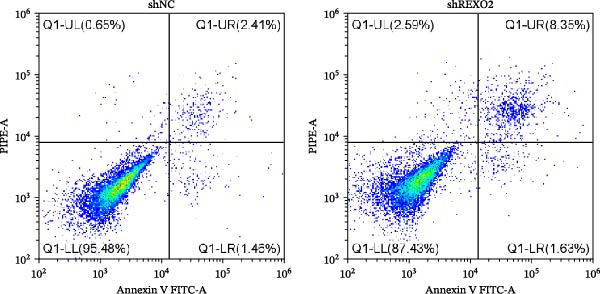
(I)

**Figure:**
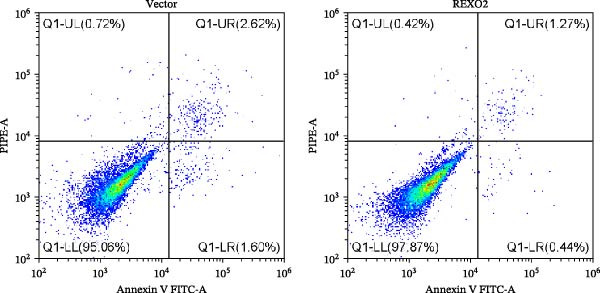
(J)

**Figure:**
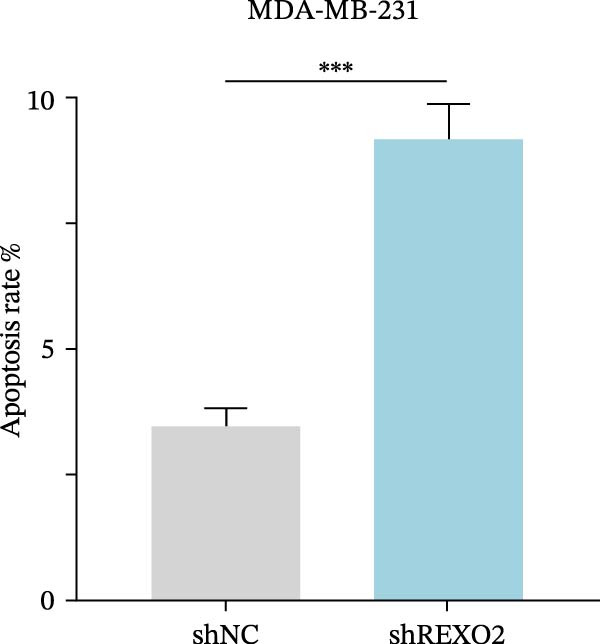
(K)

**Figure:**
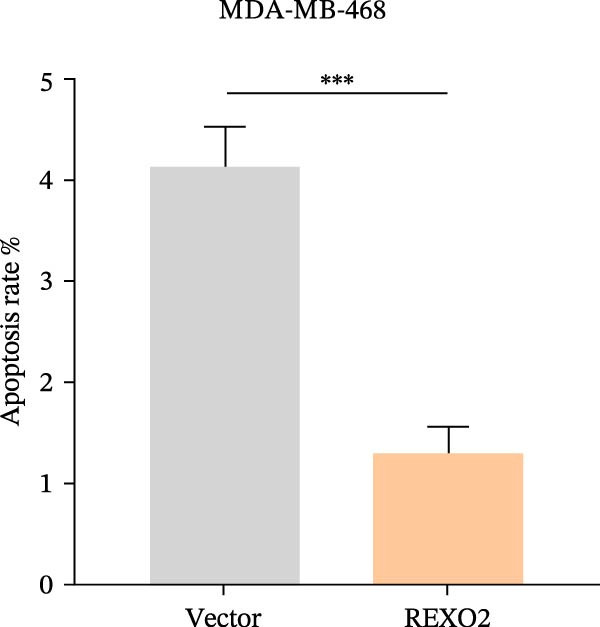
(L)

## 4. Discussion

Despite remarkable advances in the diagnosis and treatment of BC in recent years, a considerable proportion of patients still had a poor clinical outcome [1]. Recently, mitochondrial function has emerged as a key player in tumor progression and development [24, 25]. Therefore, targeting mitochondrial pathways is expected to serve as a novel therapeutic target in BC management.

Our study presents a comprehensive detection of MRPs in BC. By integrating multiomics analyses, we for the first time systematically revealed that MRPs are remarkably activated in BC. Moreover, a robust scoring system (MPAS) was developed by using machine learning framework in BC which could assess clinical outcome and therapeutic response.

Our proposed MPAS presented a robust ability on assessment of clinical outcomes across multiple BC cohorts. SHAP analysis was employed to identify the contribution of each feature to prediction prognosis, enhancing the interpretability of MPAS. The results suggested that five hub genes (REXO2, CASP9, GSTK1, HSPA9, and VDAC1) had the highest mean SHAP values, indicating their crucial roles in model predictions, with REXO2 emerging as a central factor in MPAS score system. This interpretable modeling algorithm offers may improve the potential potency of models in clinical decision‐making.

Accumulating evidence indicates that hub genes of MPAS play critical roles in tumor development and progression. CASP9, as a key regulator in the intrinsic (mitochondrial) apoptotic pathway, could activate in response to cellular stress and DNA damage, contributing to programmed cell death [26, 27]. In colorectal cancer, it was reported that silencing USP6NL could inhibit tumor malignant behavior through activation of CASP9 [28]. As revealed by Ravi et al. [29], Rhodiola crenulate could suppress BC by triggering CASP9‐mediated apoptosis. GSTK1 belongs to the mitochondrial subfamily of glutathione S‐transferases and contributes to cellular defense by catalyzing glutathione conjugation reactions [30]. Recent studies uncovered the protective role in multiple tumors including BC [31]. As a conserved member of the heat shock protein family, HSPA9 mainly localizes in mitochondria and has been reported to be involved in diverse tumor progression. For instance, HSPA9 can confer myeloma ferroptosis resistance through the USP14‐SLC7A11 axis which facilitates tumor progression [32]. Moreover, HSPA9 could be a therapeutic target in artesunate‐mediated programmed cell death in gastric cancer [33]. VDAC1 is a major pore‐forming protein located in the outer mitochondrial membrane that regulates mitochondrial‐mediated apoptosis. As suggested by Yang et al. [34], VSTM2L suppressed prostate cancer progression by targeting VDAC1 oligomerization. Moreover, it was observed that silencing VDAC1 could boost ferroptosis in lymphoma [35].

The MPAS model provides meaningful improvements in the evaluation of immunotherapy responses in BC. While TMB is commonly used to estimate potential responses to immunotherapy [36, 37], its predictive value is limited as a single genomic metric, highlighting the necessity for alternative biomarkers. Our results disclosed that patients with high TMB and low MPAS demonstrated favorable outcome, indicating a synergistic effect of genomic and immune activity. In addition, BC cases with low‐MPAS scores presented higher immune activity, including antigen presentation, T cell infiltration, and tumor cell recognition, highlighting these patients might be in a more immunoreactive status. The high‐MPAS group displayed higher enrichment of checkpoint expression, immunosuppressive cells, and higher T cell dysfunction and exclusion. Moreover, TIDE and IPS analyses suggested that the low‐MPAS group was more likely to benefit from ICB therapy. In conclusion, these data indicated that MPAS could serve as a promising tool for predicting immunotherapy efficacy.

Numerous research has disclosed that inflammation can facilitate tumor progression through promotion of angiogenesis and immune suppression [38–40]. Our analyses uncovered that high‐MPAS group presented higher inflammatory scores and upregulation of IRGs, indicating BC cases with high MPAS scores might be in chronically inflammatory microenvironment. Collectively, these findings suggest that MPAS can not only predict immune landscape but also assess inflammatory status in BC.

REXO2, a homolog of human oligoribonuclease, is in special charge of decomposing short RNA fragments less than five nucleotides. Positioned in both the cytoplasm and mitochondria, REXO2 is essential in RNA turnover and mitochondrial homeostasis. Functional research demonstrates its depletion damages mitochondrial completeness, resulting in mitochondrial DNA removal, loss of 7S DNA, downregulation of mitochondrial transcripts, and injured mitochondrial translation, finally inhibiting cell growth [41, 42]. In our study, SHAP analysis identified REXO2 as a key factor of MPAS. Emerging evidence also implicates REXO2 in tumorigenesis. In HCC, REXO2 expression is significantly upregulated and serves as an independent prognostic factor for poor survival. Silencing REXO2 suppressed proliferation, invasion, and migration of HCC cells, with transcriptome analyses suggesting regulation through TNF and NF‐κB signaling as well as associations with immune cell infiltration and immune checkpoint CTLA‐4 [43]. In addition, Wang and his colleagues reported that higher expression of REXO2 was correlated with unfavorable prognosis and tumor progression in bladder cancer [43]. Consistent with previous studies, our results demonstrated that silencing REXO2 greatly suppressed cell proliferation and induced apoptosis in BC cells, whereas REXO2 overexpression presented opposite effects. Our findings highlight its potential as a novel oncogenic driver in BC and suggest that targeting REXO2 may be a promising therapeutic strategy.

Our present research has some limitations. The MPAS was mainly constructed and evaluated using publicly available BC datasets, and its prognostic utility needs to be further validated in independent real‐world clinical cohorts. Although functional cell‐based works suggested an oncogenic role of REXO2 in BC cells, the detailed molecular mechanisms remain unclear and require additional experimental validation.

## 5. Conclusion

In summary, we thoroughly elucidated the critical role of MRPs in BC prognosis and progression. The establishment of MPAS provides a robust tool that may help with patient classification and guide personalized therapy decisions. Collectively, our work opens new avenues for future research and therapeutic development in BC.

## Author Contributions

Zizhao Guo, Heng Cao, Xiang Wang, and Jianxiu Cui designed the study and wrote the original draft. Zizhao Guo, Heng Cao, Chuqi Lei, Dongxu Ma, and Jiang Wu collected the data. Zizhao Guo, Heng Cao, Chuqi Lei, Zeyu Xing, and Chenyu Zhao analyzed the data. Zizhao Guo, Heng Cao, and Jianxiu Cui visualized the data.

## Funding

This research was funded in part by the CAMS Innovation Fund for Medical Sciences (Grants 2021‐I2M‐1‐014 and H082311‐1).

## Disclosure

All authors read and approved the final manuscript.

## Ethics Statement

The authors have nothing to report.

## Conflicts of Interest

The authors declare no conflicts of interest.

## Supporting Information

Additional supporting information can be found online in the Supporting Information section.

## Supporting information


**Supporting Information** Figure S1. The clinical relevance of top features in MPAS by using (A) HPA website and (B) GEPIA2 database. Figure S2. Function enrichment analysis by using GSEA method for (A) high‐MPAS group and (B) low‐MPAS group. Figure S3. Drug sensitivity analysis. Table S1. A total of 149 MitoPathways from MitoCarta3.0 database. Table S2. The primer sequences of each gene. Table S3. Silencing sequences of REXO2. Table S4. Univariate Cox regression analysis of mitochondria pathways‐related genes.

## Data Availability

The datasets used and/or analyzed during the current study are available from the corresponding author on reasonable request.
