# Supplementary material for: Mitochondria Pathway Signature Predicts Prognosis and Therapeutic Response and Identifies REXO2 as a Crucial Regulator in Breast Cancer
Source: Mediators Inflamm. 2026 Feb 16;2026:8994064. doi: 10.1155/mi/8994064 (PMC12910182; doi:10.1155/mi/8994064)
Supplement: Supplementary file 1 — Supporting Information Figure S1. The clinical relevance of top features in MPAS by using (A) HPA website and (B) GEPIA2 database. Figure S2. Function enrichment analysis by using GSEA method for (A) high‐MPAS group and (B) low‐MPAS group. Figure S3. Drug sensitivity analysis. Table S1. A total of 149 MitoPathways from MitoCarta3.0 database. Table S2. The primer sequences of each gene. Table S3. Silencing sequences of REXO2. Table S4. Univariate Cox regression analysis of mitochondria pathways‐related genes. [file MI-2026-8994064-s001.docx]

**Supplementary Materials**

**Supplementary methods**

The Random Survival Forest (RSF) model was implemented using the randomForestSRC package (v3.2.3). Key hyperparameters, including the number of trees (ntree) and the number of variables randomly selected at each split (mtry), were tuned using internal cross-validation, and the optimal parameter combination was selected based on the highest concordance index (C-index).

The survival support vector machine (survival-SVM) model was constructed using the survivalsvm package with a ranking-based approach. Linear, polynomial, and radial basis function (RBF) kernels were evaluated, and key hyperparameters, including kernel type and cost, were optimized through cross-validation to maximize the C-index.

Elastic Net, LASSO, and Ridge regression models were developed using the glmnet package (v4.1-8). The regularization parameter (λ) was determined by cross-validation, while the mixing parameter (α) was evaluated over a range from 0 to 1 in increments of 0.1. Stepwise Cox proportional hazards regression was performed using the survival package, with model selection guided by the Akaike Information Criterion (AIC) through both forward and backward procedures.

CoxBoost modeling was conducted using the CoxBoost package. The optimal penalty parameter was identified using the optimCoxBoostPenalty function, and the number of boosting iterations was selected based on cross-validation performance using the cv.CoxBoost function.

The partial least squares Cox (plsRcox) model was fitted using the plsRcox package, with the optimal number of components determined via cross-validation using the cv.plsRcox function. Supervised principal components (SuperPC) analysis was performed using the superpc package, in which the optimal feature selection threshold was identified through cross-validation. A pre-validation strategy was applied to reduce the risk of overfitting due to the limited sample size.

The gradient boosting machine (GBM) model was implemented using the gbm package, and the optimal number of trees was selected based on cross-validation performance using the cv.gbm function.

**Supplemental Figure**

**Supplemental Figure S1**


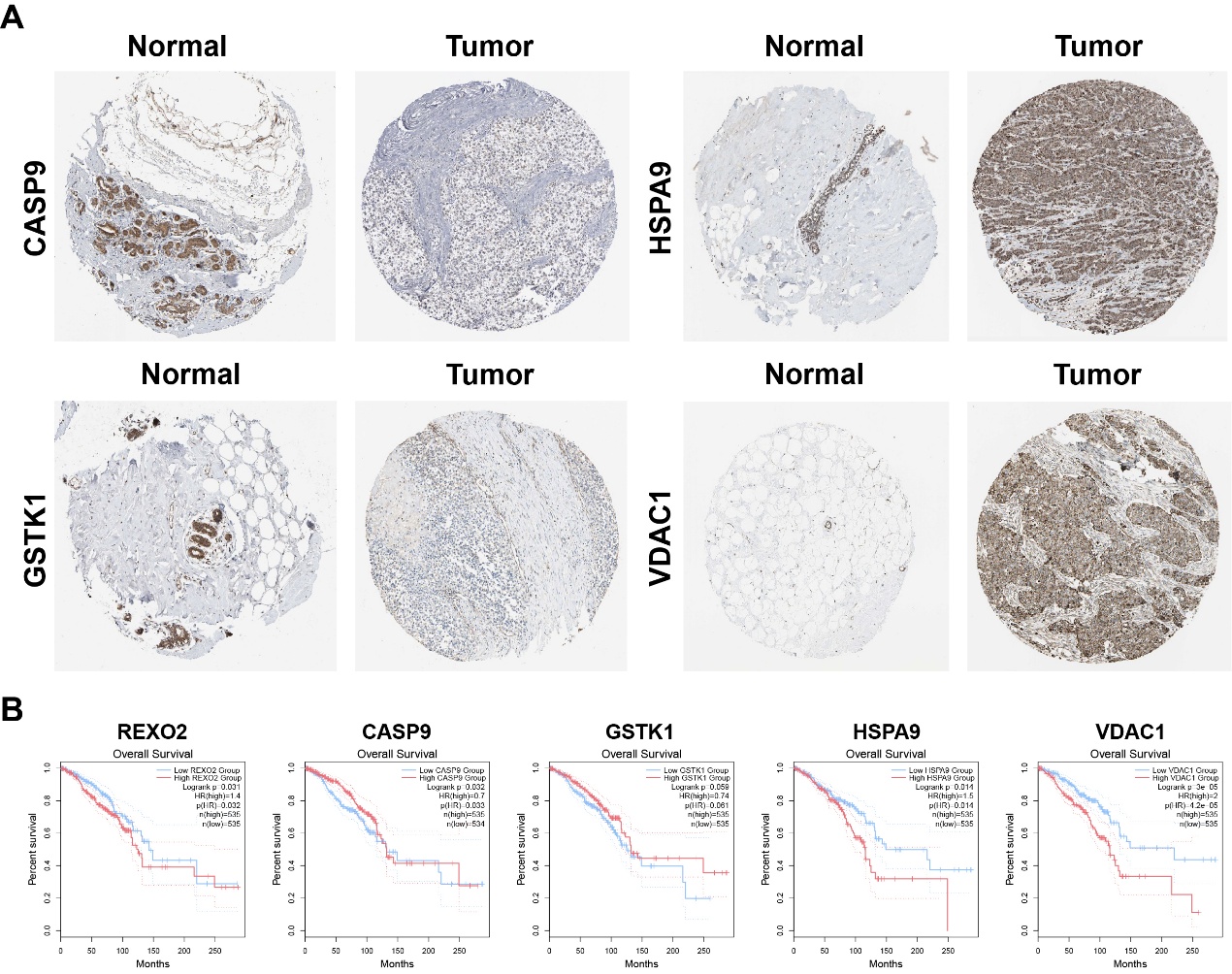


**Supplemental Figure S1.** The clinical relevance of top features in MPAS by using (A) HPA website and (B) GEPIA2 database.

**Supplemental Figure S2**


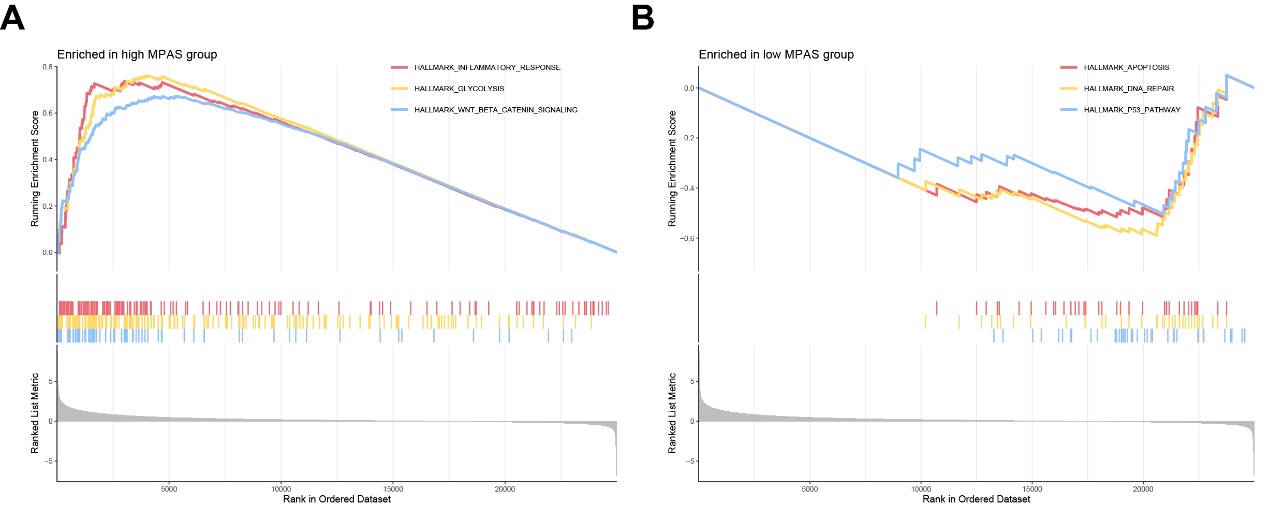


**Supplemental Figure S2.** Function enrichment analysis by using GSEA method for (A) high-MPAS group and (B) low-MPAS group.

**Supplemental Figure S3**


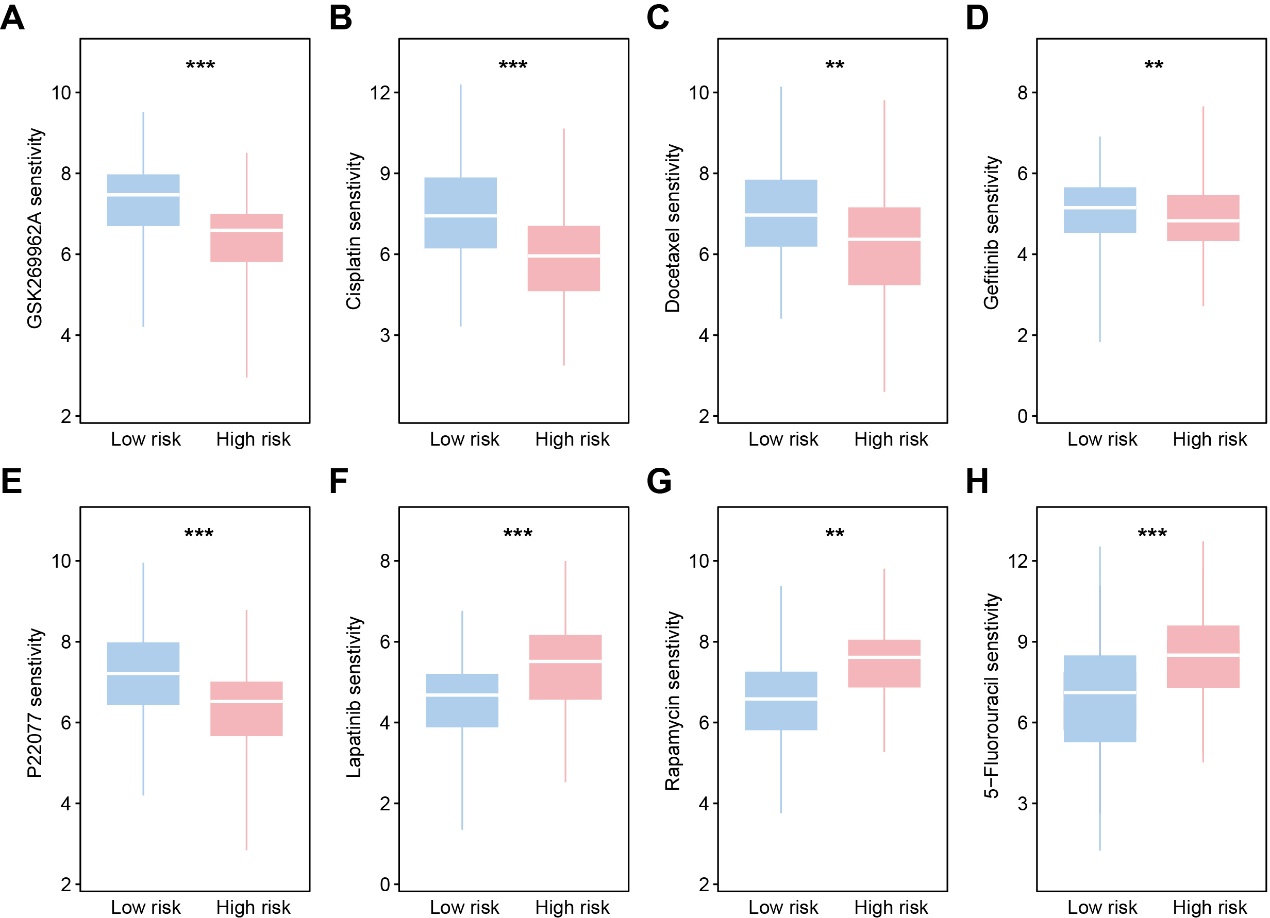


**Supplemental Figure S3.** Drug sensitivity analysis.

**Supplemental Table**

**Supplemental Table S1.** A total of 149 MitoPathways from MitoCarta3.0 database.

| **MitoPathway** |
| --- |
| Mitochondrial central dogma |
| mtDNA maintenance |
| mtDNA replication |
| mtDNA nucleoid |
| mtDNA repair |
| mtDNA modifications |
| mtDNA stability and decay |
| mtRNA metabolism |
| Transcription |
| mtRNA granules |
| Polycistronic mtRNA processing |
| mt-tRNA modifications |
| mt-rRNA modifications |
| mt-mRNA modifications |
| mtRNA stability and decay |
| Translation |
| Mitochondrial ribosome |
| Mitochondrial ribosome assembly |
| Translation factors |
| mt-tRNA synthetases |
| fMet processing |
| Protein import, sorting and homeostasis |
| Protein import and sorting |
| TOM |
| SAM |
| MIA40 |
| TIM22 carrier pathway |
| TIM23 presequence pathway |
| Import motor |
| OXA |
| Preprotein cleavage |
| Protein homeostasis |
| Proteases |
| Chaperones |
| OXPHOS |
| OXPHOS subunits |
| OXPHOS assembly factors |
| Complex I |
| CI subunits |
| CI assembly factors |
| Complex II |
| CII subunits |
| CII assembly factors |
| Complex III |
| CIII subunits |
| CIII assembly factors |
| Complex IV |
| CIV subunits |
| CIV assembly factors |
| Complex V |
| CV subunits |
| CV assembly factors |
| Respirasome assembly |
| Cytochrome C |
| Metabolism |
| Carbohydrate metabolism |
| Gluconeogenesis |
| Malate-aspartate shuttle |
| Glycerol phosphate shuttle |
| Pyruvate metabolism |
| TCA cycle |
| TCA-associated |
| Itaconate metabolism |
| Ketone metabolism |
| Propanoate metabolism |
| Lipid metabolism |
| Fatty acid oxidation |
| Type II fatty acid synthesis |
| Lipoate insertion |
| Cardiolipin synthesis |
| Cholesterol, bile acid, steroid synthesis |
| Cholesterol-associated |
| Phospholipid metabolism |
| Eicosanoid metabolism |
| Amino acid metabolism |
| Branched-chain amino acid metabolism |
| Branched-chain amino acid dehydrogenase complex |
| Lysine metabolism |
| Serine metabolism |
| Glycine metabolism |
| Glycine cleavage system |
| Glutamate metabolism |
| Proline metabolism |
| Glyoxylate metabolism |
| GABA metabolism |
| Catechol metabolism |
| Kynurenine metabolism |
| Urea cycle |
| Nucleotide metabolism |
| Nucleotide import |
| Nucleotide synthesis and processing |
| Creatine metabolism |
| Metals and cofactors |
| Carnitine synthesis and transport |
| Carnitine shuttle |
| Coenzyme A metabolism |
| Coenzyme Q metabolism |
| Copper metabolism |
| Iron homeostasis |
| Heme synthesis and processing |
| Fe-S cluster biosynthesis |
| Molybdenum cofactor synthesis and proteins |
| NAD biosynthesis and metabolism |
| Tetrahydrobiopterin synthesis |
| Fe-S-containing proteins |
| Heme-containing proteins |
| Vitamin metabolism |
| Choline and betaine metabolism |
| Biotin utilizing proteins |
| Vitamin A metabolism |
| Vitamin B1 metabolism |
| Vitamin B2 metabolism |
| Vitamin B6 metabolism |
| Vitamin B12 metabolism |
| Vitamin C metabolism |
| Vitamin D metabolism |
| Folate and 1-C metabolism |
| Detoxification |
| Xenobiotic metabolism |
| ROS and glutathione metabolism |
| Amidoxime reducing complex |
| Selenoproteins |
| Electron carriers |
| Cytochromes |
| Q-linked reactions, other |
| Sulfur metabolism |
| Small molecule transport |
| SLC25A family |
| ABC transporters |
| Sideroflexins |
| Calcium uniporter |
| Signaling |
| Calcium homeostasis |
| Calcium cycle |
| Mitochondrial permeability transition pore |
| EF hand proteins |
| Immune response |
| cAMP-PKA signaling |
| Mitochondrial dynamics and surveillance |
| Fusion |
| Fission |
| Organelle contact sites |
| Intramitochondrial membrane interactions |
| Trafficking |
| Mitophagy |
| Autophagy |
| Apoptosis |
| Cristae formation |
| MICOS complex |

**Supplemental Table S2.** The primer sequences of each gene.

| **Gene** | **Sequence** | |
| --- | --- | --- |
| REXO2 | Forward | 5′-GAACTGTGCAGACGCTGGTAT-3′ |
|  | Reverse | 5′-CATCAAGTGCCCTATGAGAAGC-3′ |
| β-Actin | Forward | 5′-CATGTACGTTGCTATCCAGGC-3′ |
|  | Reverse | 5′-CTCCTTAATGTCACGCACGAT-3′ |

**Supplemental Table S3.** Silencing sequences of REXO2.

| **Gene** | **Sequence** | |
| --- | --- | --- |
| sh-REXO2-1 |  | 5′-GCAGTGAAGGAGAGTACAATT-3′ |
| sh-REXO2-2 |  | 5′-CCGAAATAACATCTTCAAGAA-3′ |
| sh-REXO2-3 |  | 5′-GATATTGAGAAGGATCAGATT-3′ |

**Supplemental Table S4.** Univariate Cox regression analysis of mitochondria pathways-related genes.

| Gene | HR | HR.95L | HR.95H | pvalue |
| --- | --- | --- | --- | --- |
| PTRH1 | 2.251193 | 1.100248 | 4.606115 | 0.026316 |
| VDAC1 | 1.838002 | 1.299807 | 2.599043 | 0.000575 |
| REXO2 | 1.816815 | 1.264336 | 2.61071 | 0.001247 |
| APOOL | 1.750934 | 1.295178 | 2.367063 | 0.000271 |
| HSPA9 | 1.70728 | 1.223786 | 2.381793 | 0.00164 |
| HARS2 | 1.694221 | 1.087418 | 2.639634 | 0.019785 |
| CS | 1.652894 | 1.117597 | 2.444583 | 0.011843 |
| AIFM1 | 1.619444 | 1.10536 | 2.37262 | 0.01336 |
| XPNPEP3 | 1.599372 | 1.085372 | 2.356787 | 0.017591 |
| ABCB7 | 1.598091 | 1.02262 | 2.497404 | 0.039574 |
| TMEM70 | 1.583671 | 1.169049 | 2.145344 | 0.002993 |
| GRPEL1 | 1.569938 | 1.068685 | 2.306297 | 0.021534 |
| TRMT2B | 1.553657 | 1.113096 | 2.16859 | 0.009605 |
| MRPL13 | 1.552672 | 1.223745 | 1.970009 | 0.000292 |
| MRPS28 | 1.552209 | 1.131433 | 2.129472 | 0.006422 |
| PPA2 | 1.534727 | 1.077368 | 2.186243 | 0.017656 |
| NDUFS1 | 1.516679 | 1.062415 | 2.165174 | 0.02183 |
| CISD1 | 1.515633 | 1.151113 | 1.995586 | 0.003051 |
| PAICS | 1.499378 | 1.130745 | 1.988189 | 0.004901 |
| TIMM8A | 1.480541 | 1.095265 | 2.001345 | 0.010721 |
| MRPL18 | 1.472072 | 1.06614 | 2.032562 | 0.018822 |
| NDUFAB1 | 1.446259 | 1.006415 | 2.078332 | 0.046094 |
| SHMT2 | 1.443041 | 1.094755 | 1.902131 | 0.009259 |
| MRPL39 | 1.435075 | 1.021911 | 2.015282 | 0.037062 |
| YME1L1 | 1.427481 | 1.047298 | 1.945678 | 0.024295 |
| PDSS2 | 1.426869 | 1.112005 | 1.830887 | 0.005197 |
| MTFR1 | 1.425253 | 1.157459 | 1.755004 | 0.000847 |
| HINT3 | 1.416588 | 1.100722 | 1.823096 | 0.00682 |
| CHCHD4 | 1.405279 | 1.01152 | 1.952317 | 0.042535 |
| DLAT | 1.405272 | 1.049352 | 1.881914 | 0.022416 |
| ETFA | 1.400055 | 1.09497 | 1.790143 | 0.007287 |
| LACTB2 | 1.396445 | 1.094214 | 1.782154 | 0.007285 |
| SLC25A43 | 1.380884 | 1.045065 | 1.824614 | 0.023207 |
| HCCS | 1.363601 | 1.011728 | 1.837853 | 0.041697 |
| PDP1 | 1.363562 | 1.117074 | 1.66444 | 0.002302 |
| PITRM1 | 1.359652 | 1.003184 | 1.842786 | 0.047652 |
| RMDN1 | 1.348127 | 1.009774 | 1.799854 | 0.042772 |
| SLC25A5 | 1.346111 | 1.007393 | 1.798718 | 0.044456 |
| CPT1A | 1.336984 | 1.100925 | 1.623658 | 0.003389 |
| MTHFD2 | 1.306419 | 1.032123 | 1.653611 | 0.026222 |
| RTN4IP1 | 1.302436 | 1.040117 | 1.630913 | 0.021294 |
| TMEM65 | 1.296451 | 1.043229 | 1.611139 | 0.019198 |
| OXR1 | 1.288095 | 1.03171 | 1.608193 | 0.025375 |
| BNIP3 | 1.285729 | 1.026074 | 1.611091 | 0.028991 |
| QRSL1 | 1.282677 | 1.002933 | 1.640447 | 0.047334 |
| SUGCT | 1.216223 | 1.004264 | 1.472918 | 0.045122 |
| ABAT | 0.881844 | 0.778263 | 0.99921 | 0.048569 |
| C15orf48 | 0.857727 | 0.741889 | 0.991651 | 0.038151 |
| PMAIP1 | 0.850417 | 0.736282 | 0.982244 | 0.027551 |
| BCL2 | 0.848671 | 0.730399 | 0.986094 | 0.032125 |
| HSD17B8 | 0.828546 | 0.69 | 0.994911 | 0.043945 |
| CHPT1 | 0.814765 | 0.688648 | 0.963979 | 0.016962 |
| BCL2A1 | 0.810627 | 0.696109 | 0.943984 | 0.006896 |
| ACSF2 | 0.803363 | 0.679127 | 0.950326 | 0.010638 |
| CYP27A1 | 0.750359 | 0.638756 | 0.881461 | 0.000473 |
| ACADM | 0.749665 | 0.577667 | 0.972876 | 0.030253 |
| OSBPL1A | 0.746805 | 0.587784 | 0.948847 | 0.016859 |
| DNAJC4 | 0.745176 | 0.556478 | 0.99786 | 0.048343 |
| PLGRKT | 0.745086 | 0.584695 | 0.949474 | 0.017352 |
| PNKD | 0.743676 | 0.572251 | 0.966453 | 0.026747 |
| SLC25A29 | 0.737448 | 0.584247 | 0.930822 | 0.010368 |
| ECHDC2 | 0.734692 | 0.565044 | 0.955275 | 0.021361 |
| PICK1 | 0.730766 | 0.54194 | 0.985384 | 0.039735 |
| ME3 | 0.725896 | 0.578802 | 0.910372 | 0.005559 |
| AASS | 0.720264 | 0.542489 | 0.956297 | 0.023271 |
| ACAA1 | 0.710808 | 0.507778 | 0.995017 | 0.046693 |
| GSTZ1 | 0.69161 | 0.530134 | 0.902271 | 0.006567 |
| NT5DC3 | 0.67927 | 0.470162 | 0.981382 | 0.039391 |
| HSCB | 0.678214 | 0.485308 | 0.947798 | 0.02297 |
| SLC8B1 | 0.676087 | 0.482018 | 0.948293 | 0.023359 |
| SLC25A41 | 0.670517 | 0.470386 | 0.955798 | 0.027111 |
| GSTK1 | 0.663428 | 0.498624 | 0.882702 | 0.004858 |
| DNAJC30 | 0.661346 | 0.457169 | 0.956711 | 0.028173 |
| AK3 | 0.650394 | 0.472538 | 0.895192 | 0.008309 |
| NDUFB1 | 0.644811 | 0.473635 | 0.877853 | 0.00531 |
| YBEY | 0.644599 | 0.483702 | 0.859016 | 0.002725 |
| MRPL16 | 0.642015 | 0.442253 | 0.932008 | 0.019794 |
| MPV17 | 0.635389 | 0.41137 | 0.981403 | 0.040895 |
| TMEM14C | 0.621632 | 0.425139 | 0.90894 | 0.014187 |
| GRHPR | 0.600185 | 0.4069 | 0.885282 | 0.010041 |
| ADHFE1 | 0.594807 | 0.369764 | 0.956814 | 0.032195 |
| RBFA | 0.578046 | 0.374459 | 0.892321 | 0.013351 |
| COX6B2 | 0.562436 | 0.356962 | 0.886185 | 0.013107 |
| SDR39U1 | 0.543932 | 0.315939 | 0.936451 | 0.028032 |
| TRMT5 | 0.52812 | 0.318101 | 0.876801 | 0.013577 |
| CASP9 | 0.491058 | 0.316051 | 0.762972 | 0.00156 |
